# An Unexpected Function of the Prader-Willi Syndrome Imprinting Center in Maternal Imprinting in Mice

**DOI:** 10.1371/journal.pone.0034348

**Published:** 2012-04-04

**Authors:** Mei-Yi Wu, Ming Jiang, Xiaodong Zhai, Arthur L. Beaudet, Ray-Chang Wu

**Affiliations:** 1 Department of Biochemistry and Molecular Biology, George Washington University, Washington, District of Columbia, United States of America; 2 Department of Molecular and Human Genetics, Baylor College of Medicine, Houston, Texas, United States of America; CNRS, France

## Abstract

Genomic imprinting is a phenomenon that some genes are expressed differentially according to the parent of origin. Prader-Willi syndrome (PWS) and Angelman syndrome (AS) are neurobehavioral disorders caused by deficiency of imprinted gene expression from paternal and maternal chromosome 15q11–q13, respectively. Imprinted genes at the PWS/AS domain are regulated through a bipartite imprinting center, the PWS-IC and AS-IC. The PWS-IC activates paternal-specific gene expression and is responsible for the paternal imprint, whereas the AS-IC functions in the maternal imprint by allele-specific repression of the PWS-IC to prevent the paternal imprinting program. Although mouse chromosome 7C has a conserved PWS/AS imprinted domain, the mouse equivalent of the human AS-IC element has not yet been identified. Here, we suggest another dimension that the PWS-IC also functions in maternal imprinting by negatively regulating the paternally expressed imprinted genes in mice, in contrast to its known function as a positive regulator for paternal-specific gene expression. Using a mouse model carrying a 4.8-kb deletion at the PWS-IC, we demonstrated that maternal transmission of the PWS-IC deletion resulted in a maternal imprinting defect with activation of the paternally expressed imprinted genes and decreased expression of the maternally expressed imprinted gene on the maternal chromosome, accompanied by alteration of the maternal epigenotype toward a paternal state spread over the PWS/AS domain. The functional significance of this acquired paternal pattern of gene expression was demonstrated by the ability to complement PWS phenotypes by maternal inheritance of the PWS-IC deletion, which is in stark contrast to paternal inheritance of the PWS-IC deletion that resulted in the PWS phenotypes. Importantly, low levels of expression of the paternally expressed imprinted genes are sufficient to rescue postnatal lethality and growth retardation in two PWS mouse models. These findings open the opportunity for a novel approach to the treatment of PWS.

## Introduction

Genomic imprinting regulates gene expression only from one allele that is inherited either from the mother or from the father. Genomic imprinting is important as defects in this process often result in human diseases. Human chromosome region 15q11–q13 represents an imprinted domain referred as the PWS/AS domain, because paternal deletions cause Prader-Willi syndrome (PWS) and maternal deletions cause Angelman syndrome (AS) (for review [1]). The symptoms of PWS include neonatal feeding difficulties and hypotonia, morbid obesity developing in early childhood, and mild mental retardation. AS is characterized by ataxia, absence of speech, seizures, and mental retardation.

The PWS/AS imprinted domain contains a number of paternally expressed genes, including *MKRN3*, *MAGEL2*, *NDN*, *C15ORF2*, *SNURF*-*SNRPN*, and C/D box small nucleolar RNAs (snoRNAs) *SNORD107*, *SNORD64*, *SNORD108*, *SNORD109A*, *SNORD116*, *SNORD115*, and *SNORD109B* [1]. Mouse chromosome 7C has a conserved PWS/AS imprinted domain with exception of presence of *Frat3* and absence of *C15orf2*, *Snrod108*, and *Snord109a/b* [2]. *SNURF*-*SNRPN*/*Snurf*-*Snrpn* (hereafter abbreviated *SNRPN*/*Snrpn*) encodes two different proteins within a single transcript [3]. Many upstream exons of *SNRPN*/*Snrpn* were identified [4], [5], [6]. With *SNRPN*/*Snrpn* exon 1 associated with the major promoter and upstream exons with weaker promoter activity, there are alternative transcripts starting from these *SNRPN*/*Snrpn* exons and span the *UBE3A*/*Ube3a* antisense transcript [7], [8]. SnoRNAs are encoded within these large *SNRPN* sense/*UBE3A* antisense and *Snrpn* sense/*Ube3a* antisense transcripts derived from both *SNRPN*/*Snrpn* major and upstream alternative promoters. *SNORD116*/*Snord116* and *SNORD115*/*Snord115* are present as multiple copy gene clusters. Among these paternally expressed imprinted genes, *SNORD116* plays a major role in PWS etiology, because deficiency of this gene caused the key characteristics of the PWS phenotype in human [9], [10], [11]. Evidence from two mouse models with different targeted deletions of *Snord116* supports the *Snord116* function in early postnatal growth, motor learning, and feeding regulation [12], [13]. Deficiency of other genes in the PWS/AS domain may also contribute to the PWS phenotype [2], [14], [15]. On the other hand, *UBE3A* is the AS gene and encodes E6-AP ubiquitin-protein ligase expressed preferentially from the maternal chromosome in brains [16], [17]. Mutations of the *Ube3a* in mice resulted in the phenotype resembling human AS [18], [19].

Imprinted genes at the PWS/AS domain are coordinately regulated through a *cis*-acting imprinting center that contains two functional elements, the PWS-IC and AS-IC. Numerous studies in humans patients and mouse models have led to the suggestion that on the paternal chromosome, the PWS-IC is a positive regulatory element required for establishment and maintenance of paternal imprinting [6], [20], [21], whereas on the maternal chromosome, the AS-IC is suggested to function in allele-specific repression of the PWS-IC to prevent a paternal imprinting program [22], [23], [24]. However, the equivalent of the human AS-IC element has not yet been identified in mice. Three mouse models for the PWS-IC deletions have been generated by the introduction of deletions at the *Snrpn* promoter. In first model, a deletion of a 35-kb fragment at the paternal *Snrpn* promoter led to a maternal pattern of DNA methylation and gene expression on the paternal chromosome, resulting in perinatal lethality [20]. In another study, paternal inheritance of a 4.8-kb deletion (Δ4.8) at the *Snrpn* exon 1 caused a mosaic imprinting defect, resulting in partial lethality and growth retardation [6]. In the third study, a 6-kb deletion extending 1 kb further upstream of the Δ4.8 region at the *Snrpn* promoter exhibits a complete PWS-IC deletion phenotype [25]. On the other hand, attempts to define the equivalent of the human AS-IC element by targeted mutations in mice so far have been unsuccessful [26], [27]. However, an insertion/duplication mutation 13 kb upstream of *Snrpn* exon 1 resulted in an AS imprinting defect [26], and a targeted replacement of mouse PWS-IC with human PWS-IC caused a maternal imprinting defect with an AS phenotype [14].

In previous study, we have used the Δ4.8 mouse model to demonstrate the function of the PWS-IC in the regulation of paternal imprinting [6]. In current study, we used this mouse model to investigate the imprinting features on the maternal chromosome regulated by the PWS-IC. If the PWS-IC is required only for paternal imprinting and is suppressed on the maternal chromosome, then, deletion of the PWS-IC should not affect the maternal imprint. Unexpectedly, we found that maternal inheritance of the PWS-IC deletion resulted in a maternal imprinting defect accompanied by an altered maternal pattern of gene expression and epigenetic modifications, toward a paternal state. Our findings provide the first evidence that the PWS-IC is required for maternal imprinting in mice.

## Results

### Maternal inheritance of the PWS-IC Δ4.8 mutation activated the upstream alternative *Snrpn* promoter in *cis*

**Figure 1:**
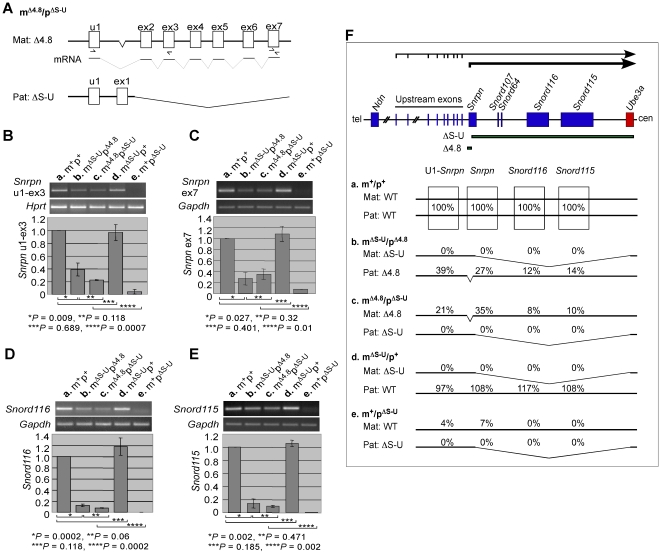
Expression analysis of *Snrpn*, *Snrod116*, and *Snord115* in mice carrying the Δ4.8 mutation and/or the ΔS-U mutation. (A) Genomic structure of the maternal Δ4.8 allele and the paternal ΔS-U allele in the m^Δ4.8^p^ΔS-U^ mice. The Δ4.8 mutation removes exon 1 of *Snrpn*. *Snrpn* is still able to transcribe from the upstream exons splicing to *Snrpn* exon 2. The relative positions of the primers specific for upstream exon 1 and exon 3 (u1-ex3) and for the downstream exon 7 (ex7) designed for RT-PCR and qRT-PCR are indicated (half-arrows). The ΔS-U mutation removes *Snrpn* from exon 2 to exon 10. (B–E) The *Snrpn* u1-ex3 (B), *Snrpn* exon 7 (C), *Snrod116* (D), and *Snord115* (E) transcripts were analyzed by RT-PCR (top) and quantitative RT-PCR (bottom). Total RNA was isolated from brains of wild-type mice (a, m^+^p^+^) (n = 5), mice inheriting the ΔS-U mutation maternally and the Δ4.8 mutation paternally (b, m^ΔS-U^p^Δ4.8^) (n = 5), mice inheriting the Δ4.8 mutation maternally and the ΔS-U mutation paternally (c, m^Δ4.8^p^ΔS-U^) (n = 5), mice with only the maternally inherited ΔS-U mutation (d, m^ΔS-U^p^+^) (n = 5), and mice with only the paternally inherited ΔS-U mutation (e, m^+^p^ΔS-U^) (n = 5). RT-PCR analyses were performed using 2.0 µg total RNA (top). For quantitative RT-PCR, the levels of gene expression from wild-type mice were set as 1 (bottom). Transcripts of *Hprt* were amplified as an endogenous control for the *Snrpn* u1-ex3 transcripts, since their sizes were similar. Transcripts of *Gapdh* were amplified as an endogenous control for the *Snrpn* exon 7, *Snrod116*, and *Snord115* transcripts. RT-PCR products: *Snrpn* u1-ex3, 295 bp; *Hprt*, 266 bp; *Snrpn* ex7, 171 bp; *Snrod116*, 98 bp; *Snrod115*, 79 bp; *Gapdh*, 97 bp. (F) Schematic representation of the mouse PWS/AS domain (top) and summary of gene expression in mice of the five different genotypes (bottom, a–e). The *Snrpn* sense/*Ube3a* antisense transcripts initiated from *Snrpn* exon 1 with the major promoter activity and from *Snrpn* upstream exons with weaker promoter activity are marked as bold and thin arrows, respectively. SnoRNAs are encoded within these large *Snrpn* sense/*Ube3a* antisense transcripts derived from both *Snrpn* major and upstream exon promoters. *Snord116* and *Snord115* are multiple copy gene clusters. The centromeric (cen) and the telomeric (tel) positions are indicated. Paternally and maternally expressed genes are marked as blue and red boxes, respectively. ΔS-U indicates a large deletion from *Snrpn* exon 2 to *Ube3a*. Δ4.8 indicates a 4.8-kb deletion at *Snrpn* exon 1. The levels of the *Snrpn* u1-ex3, *Snrpn* exon 7, *Snrod116*, and *Snord115* transcripts from wild-type mice were set as 100%. Mat, maternal chromosome; Pat, paternal chromosome.

To study the maternal imprinting features regulated by the PWS-IC, we investigated the maternal pattern of gene expression in the mouse model with the PWS-IC Δ4.8 mutation at *Snrpn* exon 1. *Snrpn* is paternally expressed from exon 1 with the major promoter and from alternative upstream exons with weaker promoter activity [6]. Although exon 1 of *Snrpn* was removed by the Δ4.8 mutation, *Snrpn* is still able to transcribe from the upstream exon promoter, initiating at alternative upstream exons splicing to *Snrpn* exon 2 [6] (Figure 1A). Using RT-PCR and quantitative RT-PCR analyses, the alternative *Snrpn* transcripts could be detected by primers specific for upstream exon 1 and exon 3 (u1-ex3) to measure the weaker promoter activity (Figure 1A and 1B), and by primers specific for the downstream exon 7 (ex7) to measure both major and weaker promoter activity (Figure 1A and 1C). The analysis of maternal-specific expression of the *Snrpn* transcripts was accomplished in mice with paternal inheritance of a deletion spanning from exon 2 of *Snrpn* to *Ube3a* (ΔS-U) [28]. Because *Snrpn* (from exon 2 to exon 10) was removed by the ΔS-U mutation on the paternal chromosome, the *Snrpn* u1-ex3 and ex7 transcripts measured, if any, could only be expressed from the maternal chromosome (Figure 1A). As the paternally expressed imprinted gene *Snrpn* was not expressed from the wild-type maternal chromosome in the m^+^p^ΔS-U^ mice (Figure 1B, 1C, and 1F, e, m^+^p^ΔS-U^), it is surprising that *Snrpn* was partially expressed from the maternal Δ4.8 chromosome in the m^Δ4.8^p^ΔS-U^ mice (Figure 1B and 1F, c, m^Δ4.8^/p^ΔS-U^, 21% of the u1-ex3 transcripts; Figure 1C and 1F, c, m^Δ4.8^/p^ΔS-U^, 35% of the ex7 transcripts), compared with that in the wild-type mice (Figure 1B, 1C, and 1F, a, m^+^/p^+^). These results suggested that maternal inheritance of the PWS-IC Δ4.8 mutation partially activated the paternally expressed imprinted gene *Snrpn* on the maternal chromosome. On the other hand, when the ΔS-U mutation was on the maternal chromosome, *Snrpn* is fully expressed from the wild-type paternal allele in the m^ΔS-U^p^+^ mice (Figure 1B and 1F, d, m^ΔS-U^/p^+^, 97% of the u1-ex3 transcripts; Figure 1C and 1F, d, m^ΔS-U^/p^+^, 108% of the ex7 transcripts), and is partially repressed on the paternal Δ4.8 chromosome in the m^ΔS-U^p^Δ4.8^ mice (Figure 1B and 1F, b, m^ΔS-U^/p^Δ4.8^, 39% of the u1-ex3 transcripts; Figure 1C and 1F, b, m^ΔS-U^/p^Δ4.8^, 27% of the ex7 transcripts), consistent with the previous report [6].

The paternally expressed imprinted genes *Snord116* and *Snord115* are encoded within the large *Snrpn* sense/*Ube3a* antisense transcripts whose expression is driven by the *Snrpn* promoter (Figure 1F, top). To further confirm activation of the *Snrpn* promoter on the maternal Δ4.8 chromosome, we examined expression of *Snord116* and *Snord115*. Since both *Snord116* and *Snord115* were also deleted by the ΔS-U mutation, maternal-specific expression of *Snord116* and *Snord115* was analyzed in mice with paternal inheritance of the ΔS-U mutation, so that the detected *Snord116* and *Snord115* transcripts could only be from the maternal chromosome. We found that *Snord116* and *Snord115* was not expressed from the maternal wild-type chromosome in the m^+^p^ΔS-U^ mice (Figure 1D–F, e, 0%), but a small amount of the *Snord116* and *Snord115* transcripts was expressed from the maternal Δ4.8 chromosome in the m^Δ4.8^p^ΔS-U^ mice (Figure 1D and 1F, c, m^Δ4.8^/p^ΔS-U^, 8% of the *Snord116* transcripts; Figures 1E and 1F, c, m^Δ4.8^/p^ΔS-U^, 10% of the *Snord115* transcripts). These suggested that maternal inheritance of the Δ4.8 mutation partially activated the *Snrpn* promoter resulting in expression of *Snord116* and *Snord115*. On the other hand, *Snord116* and *Snord115* were also partially expressed from the paternal Δ4.8 chromosome in the m^ΔS-U^p^Δ4.8^ mice (Figure 1D and 1F, b, m^ΔS-U^/p^Δ4.8^, 12% of the *Snord116* transcripts; Figure 1E and 1F, b, m^ΔS-U^/p^Δ4.8^, 14% of the *Snord115* transcripts).

Activation of the *Snrpn* promoter by maternal inheritance of the Δ4.8 mutation was demonstrated not only when paternal inheritance of the ΔS-U mutation in the m^Δ4.8^/p^ΔS-U^ mice but also when paternally inheriting the wild-type allele in the m^Δ4.8^/p^+^ mice, since there were significant increases of the *Snrpn* u1-ex3, *Snrpn* ex7, *Snord116*, and *Snord115* transcripts in the m^Δ4.8^/p^+^ mice compared with those in wild-type mice (Figure S1).

### The paternally expressed imprinted gene *Ndn* was expressed from the maternal chromosome carrying the Δ4.8 mutation

**Figure 2 pone-0034348-g002:**
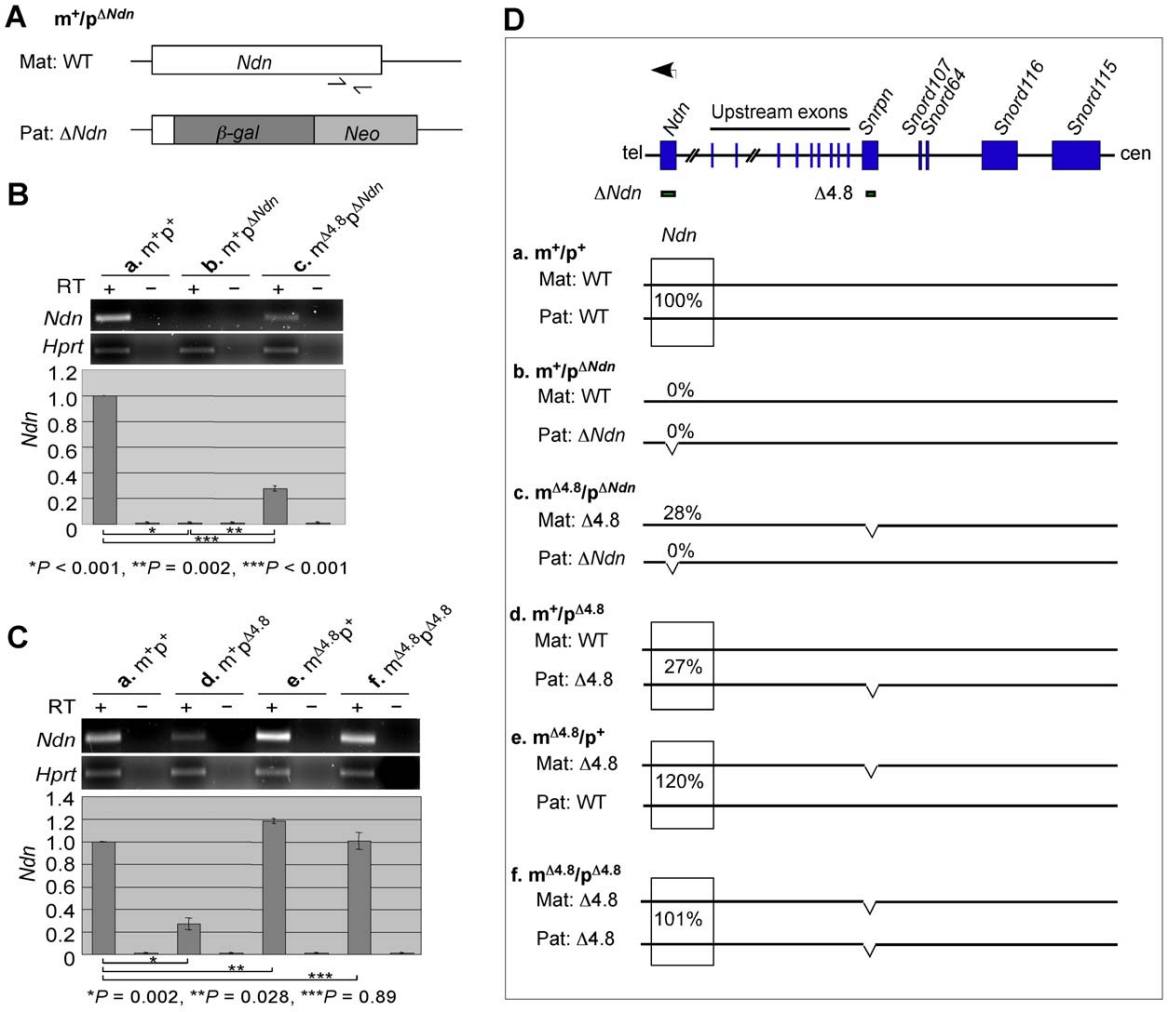
Expression analysis of the paternally expressed gene *Ndn* in mice carrying the Δ4.8 mutation and/or the Δ*Ndn* mutation. (A) Genomic structure of the maternal wild-type *Ndn* allele and the paternal Δ*Ndn* allele in the m^+^p^Δ*Ndn*^ mice. The relative position of the primer pair used for RT-PCR and qRT-PCR is indicated (half-arrows). In the Δ*Ndn* mutation, the open reading frame of *Ndn* was deleted by a replacement of *β*-galactosidase (*β*-gal) and a neomycin-resistant cassette (Neo). (B, C) The *Ndn* transcripts were analyzed by RT-PCR (top) and quantitative RT-PCR (bottom). Total RNA was isolated from brains of wild-type mice (B and C, a, m^+^p^+^) (n = 8), mice with paternal inheritance of the Δ*Ndn* mutation (B, b, m^+^p^Δ*Ndn*^) (n = 3), mice inheriting the Δ4.8 mutation maternally and the Δ*Ndn* mutation paternally (B, c, m^Δ4.8^p^Δ*Ndn*^) (n = 3), mice with paternal inheritance of the Δ4.8 mutation (C, d, m^+^p^Δ4.8^) (n = 5), mice with maternal inheritance of the Δ4.8 mutation (C, e, m^Δ4.8^p^+^) (n = 5), and mice with the Δ4.8 mutation from both the parents (C, f, m^Δ4.8^p^Δ4.8^) (n = 5). RT-PCR analyses were performed using 2.0 µg total RNA with (+) and without (−) reverse transcriptase (RT). For quantitative RT-PCR, the levels of gene expression from wild-type mice were set as 1. Transcripts of *Hprt* were amplified as an endogenous control. RT-PCR products: *Ndn*, 365 bp; *Hprt*, 266 bp. (D) Schematic representation of the mouse PWS/AS domain (top) and summary of *Ndn* expression in mice of the six different genotypes (bottom, a–f). The *Ndn* transcripts are marked as an arrow. The centromeric (cen) and the telomeric (tel) positions are indicated. Paternally expressed imprinted genes are marked as blue boxes. Δ*Ndn* indicates a deletion at *Ndn*. Δ4.8 indicates a 4.8-kb deletion at *Snrpn* exon 1. The level of the *Ndn* transcripts from wild-type mice was set as 100%. Mat, maternal chromosome; Pat, paternal chromosome.

The PWS-IC plays a dual role as the *Snrpn* promoter and as an IC in the PWS/AS region [6]. Given that the PWS-IC Δ4.8 mutation affected the maternal imprinting of the adjacent *Snrpn* upstream promoter (Figure 1), we investigated whether the Δ4.8 mutation also perturbs its IC function in maternal imprinting. Transcription of a paternally expressed imprinted gene *Ndn* was analyzed as an index of the extent of any affected gene expression within the PWS/AS region, since the *Ndn* locus is located about 1 Mb upstream of the *Snrpn* promoter. Maternal-specific expression of the *Ndn* transcripts was analyzed in mice with paternal inheritance of a deletion on *Ndn* (Δ*Ndn*) [29]. In these mice, any detected *Ndn* transcripts were expressed exclusively from the maternal chromosome and not from the paternal Δ*Ndn* chromosome, since the primer pair used for RT-PCR and qRT-PCR analyses is located at the region deleted in the Δ*Ndn* mutation (Figure 2A). Our results showed that *Ndn* was not transcribed from the maternal wild-type chromosome in the m^+^p^Δ*Ndn*^ mice (Figure 2B and 2D, b), but was partially expressed from the maternal Δ4.8 chromosome in the m^Δ4.8^p^Δ*Ndn*^ mice (Figure 2B and 2D, c, 28%). These results suggested that maternal inheritance of the Δ4.8 mutation partially activated the paternally expressed imprinted gene *Ndn* on the maternal chromosome. Therefore, the maternal PWS-IC Δ4.8 mutation disturbed not only the maternal imprint of the adjacent *Snrpn* upstream promoter but also the maternal imprint of the *Ndn* promoter which is 1 Mb away from the Δ4.8 region.

In addition, the abundance of the *Ndn* transcripts was reduced when the Δ4.8 mutation was on the paternal chromosome (Figure 2C and 2D, d, m^+^/p^Δ4.8^, 27%), as previously reported [6]. Importantly, mice with the Δ4.8 mutation on both maternal and paternal chromosomes expressed a level of the *Ndn* transcripts comparable with that in the wild-type controls (Figure 2C and 2D, a, m^+^/p^+^, 100%; f, m^Δ4.8^/p^Δ4.8^, 101%). These results suggested that maternal inheritance of the Δ4.8 mutation compensated the loss of *Ndn* expression due to paternal inheritance of the Δ4.8 mutation. Accordingly, we found a small but consistent increase in *Ndn* expression in mice with only the maternally inherited Δ4.8 mutation (Figure 2C and 2D, e, m^Δ4.8^/p^+^, 120%).

### Expression of the maternally expressed imprinted gene *Ube3a* was reduced when the Δ4.8 mutation was on the maternal chromosome

**Figure 3:**
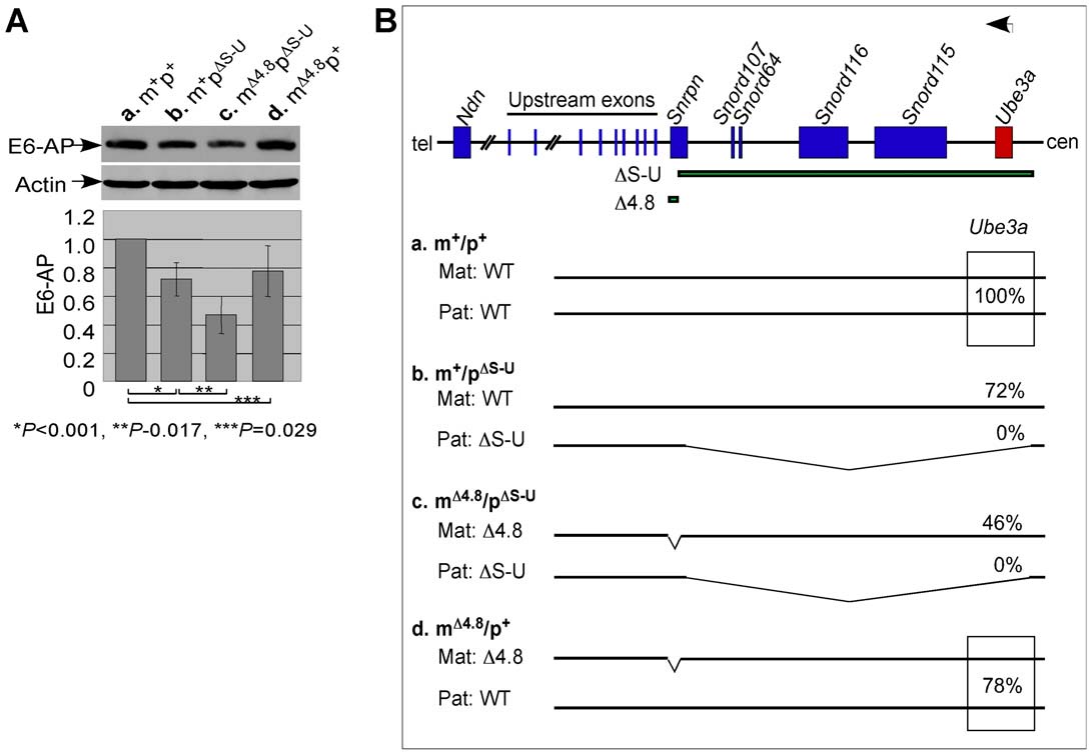
Expression analysis of the maternally expressed gene *Ube3a* in mice carrying the Δ4.8 mutation and/or the ΔS-U mutation. (A) Protein was extracted from the brains of wild-type mice (a, m^+^p^+^) (n = 5), mice with only the paternally inherited ΔS-U mutation (b, m^+^p^ΔS-U^) (n = 5), mice inheriting the Δ4.8 mutation maternally and the ΔS-U mutation paternally (c, m^Δ4.8^p^ΔS-U^) (n = 5), and mice with only maternal inheritance of the Δ4.8 mutation (d, m^Δ4.8^p^+^) (n = 5). Western blot analysis was performed using antibodies against E6-AP or actin. Expression was quantitated by densitometry. The level of E6-AP was normalized against the level of actin in each sample. The normalized level of E6-AP from the wild-type mouse was set as 1. E6-AP, ∼110 kDa; actin, ∼45 kDa. (B) Schematic representation of the mouse PWS/AS domain (top) and summary of *Ube3a* expression in mice of the four different genotypes (bottom, a–d). Expression of *Ube3a* is represented by an arrow. The centromeric (cen) and the telomeric (tel) positions are indicated. Paternally and maternally expressed genes are shown as blue and red boxes, respectively. ΔS-U indicates a large deletion from *Snrpn* exon 2 to *Ube3a*. Δ4.8 indicates a 4.8-kb deletion at *Snrpn* exon 1. The level of E6-AP in wild-type mice was set as 100%. Mat, maternal chromosome; Pat, paternal chromosome.

While the paternally expressed imprinted genes were activated, it was of interest to examine whether the maternally expressed imprinted gene was repressed. *Ube3a*, known as the AS gene, encodes E6-AP ubiquitin-protein ligase whose expression derives preferentially from the maternal chromosome in brains [16], [17], and is negatively regulated by the paternal expressed *Snrpn* sense/*Ube3a* antisense transcripts derived from the *Snrpn* promoter [14], [30], [31]. Since the maternal *Snrpn* promoter was activated by maternal inheritance of the Δ4.8 mutation (Figure 1), we found a decreased level of E6-AP in the m^Δ4.8^p^+^ mice (Figure 3A and 3B, d, 78% of E6-AP), compared with that in wild-type mice (Figure 3A and 3B, a, 100% of E6-AP). Maternal-specific reduction of *Ube3a* expression by maternal inheritance of the Δ4.8 mutation was further analyzed in mice with paternal inheritance of the ΔS-U mutation. Because *Ube3a* was deleted by the ΔS-U mutation on the paternal chromosome, *Ube3a* could only express from the maternal chromosome. Compared to the maternal wild-type chromosome in the m^+^p^ΔS-U^ mice (Figure 3A and 3B, b, 72% of E6-AP), the maternal Δ4.8 chromosome expressed a reduced level of E6-AP in the m^Δ4.8^p^ΔS-U^ mice (Figure 3A and 3B, c, 46% of E6-AP). These results suggested that maternal expression of *Ube3a* was partially repressed by maternal inheritance of the Δ4.8 mutation.

Together, we found that maternal inheritance of the PWS-IC Δ4.8 mutation changed the maternal pattern of gene expression toward a paternal state: the paternally expressed imprinted genes were partially activated and the maternally expressed imprinted genes were partially repressed on the maternal Δ4.8 chromosome.

### Maternal inheritance of the Δ4.8 mutation complemented a postnatal lethality phenotype in PWS mouse models paternally inheriting the Δ4.8 or ΔS-U mutations

The functional significance of the acquired paternal pattern of gene expression on the maternal chromosome was further investigated by genetic complementation experiments in two PWS mouse models paternally inheriting the Δ4.8 or ΔS-U mutations. The Δ4.8 mutation did not affect survival when inherited maternally (Table 1, mating II), but caused postnatal lethality in 56% of the mice when inherited paternally (Table 1, mating III), as previously reported [6]. Interestingly, there was an almost complete rescue of the lethality in mice inheriting the Δ4.8 mutation from both parents, with 96% survival rate for mice observed up to 2 months of age (Table 1, mating IV). In addition, while all mice with only paternal inheritance of the ΔS-U mutation died (Table 1, mating V), double heterozygous mice inheriting the ΔS-U mutation paternally and the Δ4.8 mutation maternally survived close to the expected Mendelian ratios (Table 1, mating VI). These results suggested that maternal inheritance of the Δ4.8 mutation complemented the lethality phenotype in the PWS mouse models paternally inheriting the Δ4.8 or ΔS-U mutations.

**Table 1:** Maternal inheritance of the Δ4.8 mutation rescued lethality caused by paternal inheritance of the Δ4.8 or ΔS-U mutations.

	Parental genotypes		Offspring			
	Female	Male	Total # of born pups	Total # of dead pups	Total # of survivors	Offspring genotypes {# of survivors (survival rate)}
I	+/+	+/+	28^a = 2^	0	28	+/+{28 (100%)}
II	Δ4.8/Δ4.8	+/+	42^b = 3^	0	42	Δ4.8/+{42 (100%)}
III	+/+	Δ4.8/Δ4.8	48^c = 3^	21	27	+/Δ4.8 {27 (56%)}
IV	Δ4.8/Δ4.8	Δ4.8/Δ4.8	95^d = 6^	4	91	Δ4.8/Δ4.8 {91 (96%)}
V	+/+	ΔS-U/+	56^e = 4^	35	21	+/+{21}; +/ΔS-U {0 (0%)}
VI	Δ4.8/Δ4.8	ΔS-U/+	53^f = 3^	1	52	Δ4.8/+{27}; Δ4.8/ΔS-U {25 (96%)}

The numbers of breeding cages are indicated by a, b, c, d, e, f, and g.

Survival offspring were observed up to 2 months of age.

### Maternal inheritance of the Δ4.8 mutation complemented a growth retardation phenotype in PWS mouse models paternally inheriting the Δ4.8 or ΔS-U mutations

**Figure 4:**
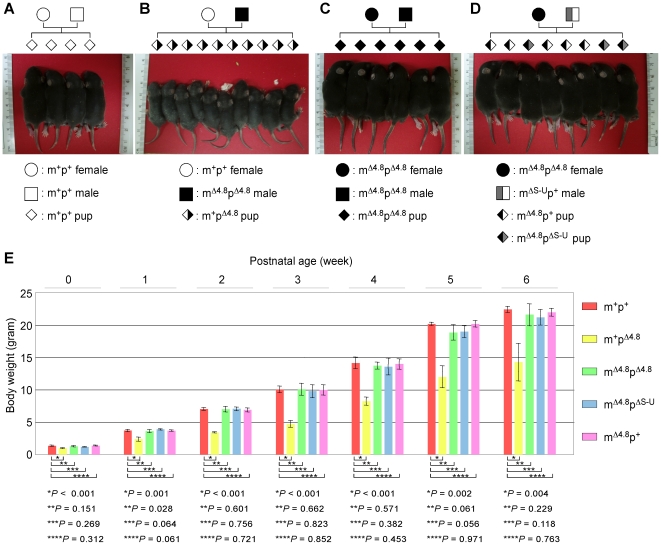
Rescue of growth retardation in the PWS mouse models by maternal inheritance of the Δ4.8 mutation. (A) Wild-type offspring were obtained from mating a wild-type female with a wild-type male. (B) Growth retardation was observed in the m^+^p^Δ4.8^ offspring obtained from mating a wild-type female with a male carrying the Δ4.8 mutation. (C) Growth retardation was rescued in the m^Δ4.8^p^Δ4.8^ offspring inheriting the Δ4.8 mutation from both the parents. (D) Double heterozygous m^Δ4.8^p^ΔS-U^ pups attained a normal body weight indistinguishable from the m^Δ4.8^p^+^ littermates. All photographs were taken when groups of litters were 10 days of age. (E) The growth retardation was analyzed by weighting groups of mice with those five different genotypes up to 6 weeks of age. m^+^p^+^, n = 6; m^+^p^Δ4.8^, n = 4; m^Δ4.8^p^Δ4.8^, n = 5; m^Δ4.8^p^ΔS-U^, n = 5; m^Δ4.8^p^+^, n = 5.

Paternal inheritance of the Δ4.8 or ΔS-U mutations resulted in not only postnatal lethality but also growth retardation in surviving mice [6], [28]. The heterozygous pups paternally inheriting the Δ4.8 mutation were smaller compared to the age-matched wild-type mice (Figure 4A, m^+^p^+^ and Figure 4B, m^+^p^Δ4.8^), as previously reported [6]. Interestingly, homozygous pups inheriting the Δ4.8 mutation from both parents had an average body size indistinguishable from age-matched wild-type mice (Figure 4A, m^+^p^+^, and Figure 4C, m^Δ4.8^p^Δ4.8^). Furthermore, the m^Δ4.8^p^ΔS-U^ double heterozygous pups obtained by mating female mice carrying the Δ4.8 mutation to male mice carrying the ΔS-U mutation have body size similar to age-matched wild-type littermates and littermates with only the maternally inherited Δ4.8 mutation (m^Δ4.8^p^+^) (Figure 4A and 4D). Measurements of body weight from groups of mice with those five different genotypes for up to 6 weeks clearly showed that maternal inheritance of the Δ4.8 mutation complements a growth retardation phenotype caused by paternal inheritance of the Δ4.8 or ΔS-U mutations (Figure 4E).

Notably, the maternal Δ4.8 chromosome expressed low levels of the paternally expressed imprinted genes *Snrpn* (21%–35%), *Snord116* (8%), *Snord115* (10%), and *Ndn* (28%) (Figure 1 and 2), which were however sufficient to complement postnatal lethality and growth retardation phenotypes in the mouse models of PWS.

### Maternal inheritance of the Δ4.8 mutation altered histone modifications at the *Snrpn* and *Ndn* promoters

**Figure 5:**
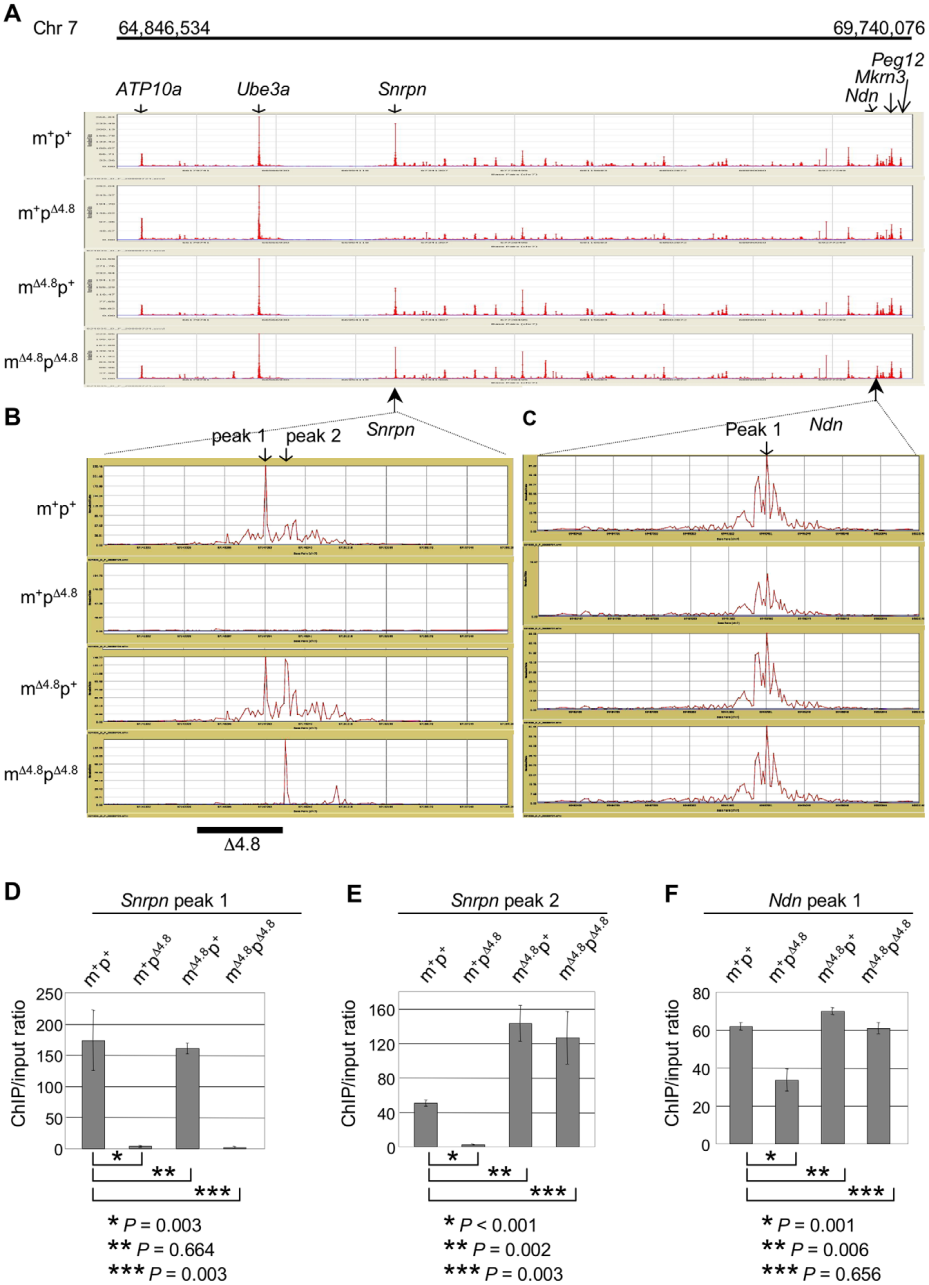
Distribution of H3K4me3 at the PWS/AS domain determined by ChIP-on-chip analysis. (A) The ChIP-on-chip profiles of H3K4me3 enrichment at the PWS/AS domain. H3K4me3 enriched peaks at gene loci are marked by arrows. *X*-axis represents the PWS/AS region (Chr7: 64,846,543–69,740,076). *Y*-axis represents normalized ChIP/input ratios. (B,C) Zoom in views of the *Snrpn* (B) and *Ndn* (C) loci. The region of the Δ4.8 mutation at the *Snrpn* promoter is indicated as a black box. (D–F) Quantification of H3K4me3 enriched peaks at *Snrpn* (D, peak 1; E, peak 2) and *Ndn* (F, peak 1) by using three mice for each genotype to perform the ChIP-on-chip experiments. Figure 5A–C showed H3K4me3 enriched peaks from one set of mice including four different genotypes.

Parent-of-origin specific epigenetic modifications on the PWS-IC correlate with transcriptional status and parent-of-origin specific epigenotypes of the imprinted genes spread over the PWS/AS domain. Given the paternal pattern of gene expression on the maternal Δ4.8 chromosome (Figures 1–3), we determined whether epigenetic status at the PWS/AS domain was altered by maternal inheritance of the Δ4.8 mutation. First, we analyzed histone modification profiles within the PWS/AS imprinted domain by ChIP-on-chip assays that combined chromatin immunoprecipitation to identify regions enriched with trimethylation on histone H3 lysine 4 (H3K4me3) and mouse genomic tiling array including sequences of the PWS/AS region from *Peg12* to *ATP10A* (Chr7: 64,846,543–69,740,076) (Figure 5A). Within the *Snrpn* locus, wild-type mice displayed a cluster of ChIP peaks surrounding the *Snrpn* promoter with the highest H3K4me3 enrichment located within a region corresponds to the Δ4.8 mutation (Figure 5B and 5D, m^+^p^+^, *Snrpn* peak 1). Surprisingly, while paternal inheritance of the Δ4.8 mutation resulted in disappearance of the entire ChIP cluster (Figure 5B, m^+^p^Δ4.8^), maternal inheritance of the Δ4.8 mutation in the m^Δ4.8^p^Δ4.8^ mice partially restored H3K4me3 modification with the highest peak shifted to the right upstream of the Δ4.8 region (Figure 5B and 5E, m^Δ4.8^p^Δ4.8^, *Snrpn* peak 2). Accordingly, the m^Δ4.8^p^+^ mice had a combinatorial pattern of the m^Δ4.8^p^Δ4.8^ and wild-type mice showing two ChIP peaks with high H3K4me3 enrichment (Figure 5B, 5D, and 5E, m^Δ4.8^p^+^, *Snrpn* peak 1 and 2). Notably, when maternal inheritance of the Δ4.8 mutation, the acquired H3K4me3 enrichment (*Snrpn* peak 2) was located within 1 kb further upstream of the Δ4.8 region (Figure 5B, m^Δ4.8^p^Δ4.8^ and m^Δ4.8^p^+^, *Snrpn* peak 2), where the full PWS-IC functional element must include with the Δ4.8 region [25].

Within the *Ndn* locus, paternal inheritance of the Δ4.8 mutation in the m^+^p^Δ4.8^ mice reduced overall amplitude of H3K4me3 by half, whereas maternal inheritance of the Δ4.8 mutation in the m^Δ4.8^p^Δ4.8^ mice restored the amplitude of H3K4me3 to the extent comparable with that in wild-type mice (Figure 5C and 5F), suggesting that maternal inheritance of the Δ4.8 mutation increased H3K4me3 at *Ndn*. Since H3K4me3 is a mark for an active state of gene expression, these findings are consistent with the results that expression of *Ndn* was partially inhibited in the m^+^p^Δ4.8^ mice, but was restored to a normal level in the m^Δ4.8^p^Δ4.8^ mice (Figure 2C), due to activation of *Ndn* on the maternal Δ4.8 chromosome (Figure 2B).

**Figure 6:**
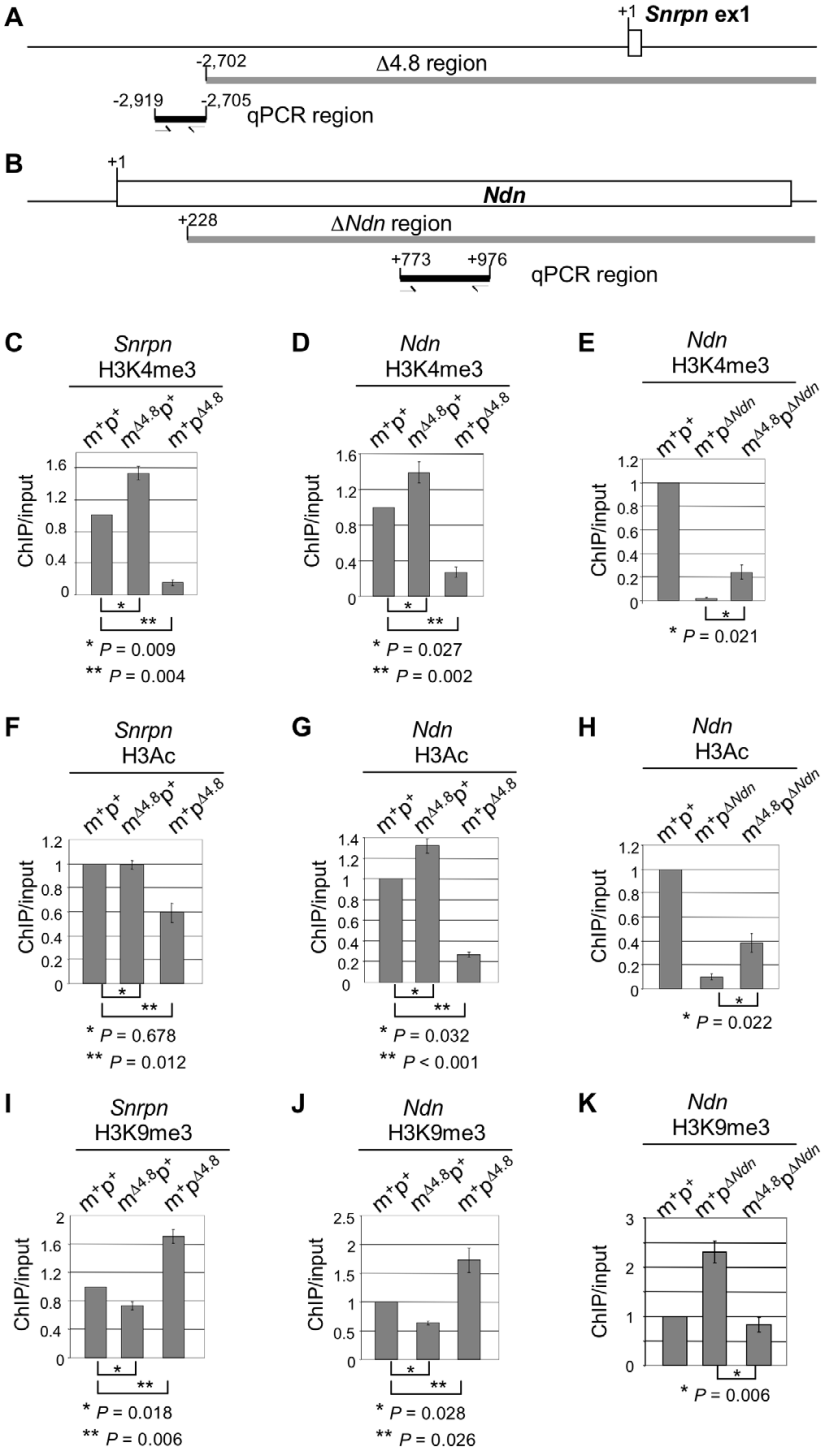
ChIP-qPCR analyses for H3K4me3, H3Ac, and H3K4me3 at *Snrpn* and *Ndn*. (A) Schematic diagram of the *Snrpn* promoter. Gene structure is shown at the top, where the white box represents the *Snrpn* exon 1 with the +1 as the major transcriptional start site. The region deleted in the Δ4.8 mutation started from −2,702 is indicated as a gray line. A primer pair (half-arrows) was used for qPCR to amplify the *Snrpn* promoter from −2,919 to −2,705 right upstream of the Δ4.8 region (black box). This qPCR region includes the *Snrpn* peak 2 region shown in Figure 5B. (B) Schematic diagram of *Ndn*. The gene structure is shown at the top, where the white box represents the *Ndn* exon with the +1 as the transcriptional start site. The region deleted in the Δ*Ndn* mutation started from +228 is indicated (gray line). A primer pair (half-arrows) was used for qPCR to amplify the region from +773 to +976 (black line). This qPCR region includes the *Ndn* peak 1 region shown in Figure 5C. (C–E) Quantification of H3K4me3 at *Snrpn* (C) and *Ndn* (D, E) in the wild-type m^+^p^+^ mice (C–E), the m^Δ4.8^p^+^ mice (C, D), the m^+^p^Δ4.8^ mice(C, D), the m^+^p^Δ*Ndn*^ mice (E), and the m^Δ4.8^p^Δ*Ndn*^ mice (E). (F–H) Quantification of H3Ac at *Snrpn* (F) and *Ndn* (G, H) in the wild-type m^+^p^+^ mice (F–H), the m^Δ4.8^p^+^ mice (F, G), the m^+^p^Δ4.8^ mice (F, G), the m^+^p^Δ*Ndn*^ mice (H), and the m^Δ4.8^p^Δ*Ndn*^ mice (H). (I–H) Quantification of H3K9me3 at *Snrpn* (I) and *Ndn* (J, K) in the wild-type m^+^p^+^ mice (I–H), the m^Δ4.8^p^+^ mice (I, J), the m^+^p^Δ4.8^ mice(I, J), the m^+^p^Δ*Ndn*^ mice (K), and the m^Δ4.8^p^Δ*Ndn*^ mice (K). The level of ChIP was normalized against the level of input in each sample. The normalized level of ChIP from the wild-type mouse was set as 1. m^+^p^+^, n = 6; m^+^p^Δ4.8^, n = 4; m^+^p^Δ4.8^, n = 4; m^+^p^Δ*Ndn*^, n = 3; m^Δ4.8^p^Δ*Ndn*^, n = 3.

The alteration of H3K4me3 modification was further confirmed by ChIP combined with quantitative PCR analysis (ChIP-qPCR). We designed a primer pair to amplify the *Snrpn* promoter right upstream of the Δ4.8 region for qPCR (Figure 6A). The qPCR fragment includes the *Snrpn* peak 2 region shown in Figure 5B. Another primer pair was designed to amplify the *Ndn* locus including the *Ndn* peak 1 region shown in Figure 5C (Figure 6B). ChIP-qPCR analyses showed a marked reduction of H3K4me3 at both *Snrpn* and *Ndn* in the m^+^p^Δ4.8^ mice when compared with those in wile-type mice, whereas the m^Δ4.8^p^+^ mice had significant increases in H3K4me3 (Figure 6C and 6D). These results support ChIP-on-chip analyses which showed reductions of H3K4me3 enrichment at *Snrpn* and *Ndn* when paternal inheritance of the Δ4.8 mutation and increases of H3K4me3 when maternal inheritance of the Δ4.8 mutation (Figure 5). To determine whether the effect of maternal inheritance of the Δ4.8 mutation is on the maternal allele, we used mice with paternal inheritance of the Δ*Ndn* mutation. In this assay, only the wild-type *Ndn* allele on the maternal chromosome could be detected because the primer pair designed for ChIP-qPCR is located at the region deleted in the Δ*Ndn* mutation (Figure 6B). Compared with wild-type mice, the m^+^p^Δ*Ndn*^ mice showed a dramatic reduction of H3K4me3 (Figure 6E). This result clearly indicates paternal-specific H3K4me3 at *Ndn* and paternal deletion contributes to the significant reduction of H3K4me3. This is similar to the human *NDN* promoter showing paternal-specific association with H3K4me3 [32]. When maternally inheriting the Δ4.8 mutation, an elevated level of H3K4me3 was detected in the m^Δ4.8^p^Δ*Ndn*^ mice compared with the m^+^p^Δ*Ndn*^ mice (Figure 6E). Since the paternal copy of *Ndn* was deleted in these mice, the elevated H3K4me3 was derived from the remaining maternal copy of *Ndn* in the m^Δ4.8^p^Δ*Ndn*^ mice.

We next examined acetylation of histone 3 (H3Ac) as an additional marker of an active gene expression state. ChIP-qPCR analyses showed reductions of H3Ac at both *Snrpn* and *Ndn* in the m^+^p^Δ4.8^ mice (Figure 6F and 6G, respectively). On the other hand, maternal inheritance of the Δ4.8 mutation did not affect H3Ac at the *Snrpn* promoter in the m^Δ4.8^p^+^ mice (Figure 6F), but did increase H3Ac at *Ndn* (Figure 6G). When the paternal *Ndn* was deleted in the m^+^p^Δ*Ndn*^ mice, a marked reduction of H3Ac was observed (Figure 6H), suggesting the paternal copy is the one preferentially modified with H3Ac. This is similar to the human *NDN* promoter showing paternal bias with H3Ac [32]. Similar to increased H3K4me3 (Figure 6E), m^Δ4.8^p^Δ*Ndn*^ mice showed an increase of H3Ac on the maternal copy of *Ndn* compared to the m^+^p^Δ*Ndn*^ mice (Figure 6H).

Finally, we examined H3K9me3 which is a mark of a repressive chromatin state. ChIP-qPCR analyses showed that the m^+^p^Δ4.8^ mice had marked increases in H3K9me3 at both *Snrpn* and *Ndn*, whereas the m^Δ4.8^p^+^ mice showed reductions of H3K9me3 compared with wild-type mice (Figure 6I and 6J). Furthermore, the m^Δ4.8^p^Δ*Ndn*^ mice showed the reduction of H3K9me3 on the maternal copy of *Ndn* when compared with the m^+^p^Δ*Ndn*^ mice (Figure 6K). There was approximately 2-fold enrichment of H3K9me3 in the m^+^p^Δ*Ndn*^ mice compared with wild-type mice (Figure 6K). It should be noted that in ChIP-qPCR analysis, the level of ChIP was normalized against the level of input in each sample: ChIP from the m^+^p^Δ*Ndn*^ mice was normalized against the input with only one copy of the maternal *Ndn* allele, while ChIP from the m^+^p^+^ mice was normalized against the input with two *Ndn* copies from both parents. It is possible that the maternal copy of *Ndn* could be preferentially modified with H3K9me3, which is similar to the human *NDN* promoter with H3K9me3 towards maternal bias [32]. Therefore, after normalization with input, the ChIP-qPCR result might show a 2-fold enrichment of H3K9me3 in the m^+^p^Δ*Ndn*^ mice compared with the m^+^p^+^ mice, even though both mice could have similar levels of H3K9me3 enrichment on their maternal wild-type copies of *Ndn*. However, we can not rule out the possibility that paternal inheritance of the Δ*Ndn* mutation acts *in trans* to increase H3K9me3 on the maternal chromosome.

### Maternal inheritance of the Δ4.8 mutation altered DNA modification at *Ndn* and *Mkrn3*

**Figure 7:**
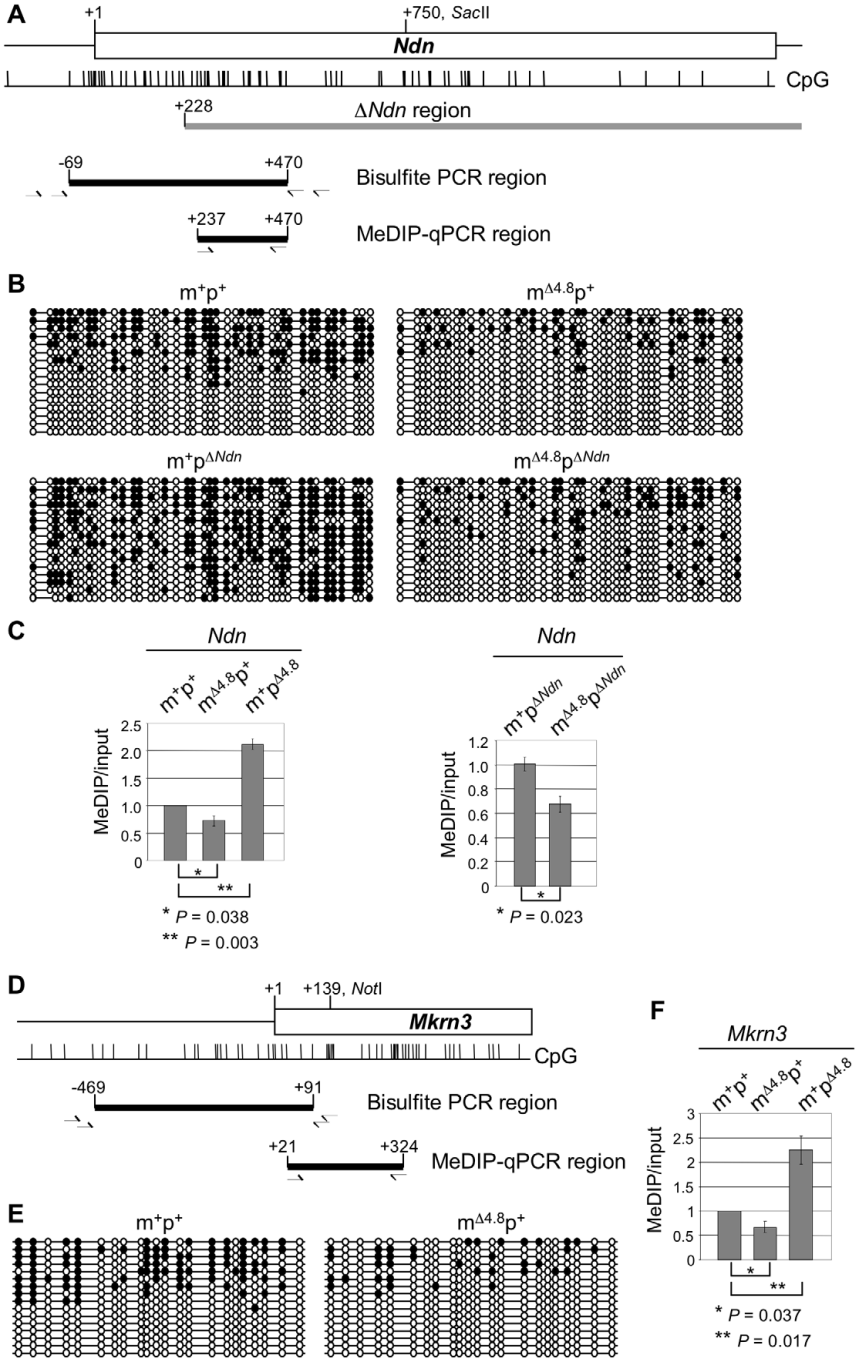
DNA methylation analyses at *Ndn* and *Mkrn3*. (A) Schematic diagram of *Ndn*. Gene structure is shown at the top, where the white box represents the *Ndn* exon with the +1 as the transcriptional start site. A *Sac*II site at +750 for Southern blot analysis in Figure S1 is indicated. Locations of CpG dinucleotides are shown as vertical bars. The region deleted in the Δ*Ndn* mutation started from +228 is indicated (gray line). Two primer pairs (half-arrows) were used for nested PCR to amplify the bisulfite-treated DNA at the *Ndn* promoter from −69 to +470 (black line). A third primer pair (half-arrows) was used for the MeDIP-qPCR analysis to amplify the region from +237 to +470 (black line). (B) Sodium bisulfite sequencing analyses of methylation status of 42 CpG dinucleotides across the *Ndn* promoter (−69 to +470) in the wild-type m^+^p^+^ mice, the m^Δ4.8^p^+^ mice, the m^+^p^Δ*Ndn*^ mice, and the m^Δ4.8^p^Δ*Ndn*^ mice. Each line represents an individual clone with open and closed circles corresponding to unmethylated and methylated CpGs, respectively. (C) MeDIP-qPCR analyses of DNA methylation at the *Ndn* promoter (+237 to +470) in the wild-type m^+^p^+^ mice, the m^Δ4.8^p^+^ mice, and the m^+^p^Δ4.8^ mice (left), as well as in the m^+^p^Δ*Ndn*^ mice and the m^Δ4.8^p^Δ*Ndn*^ mice (right). The level of MeDIP DNA was normalized against the level of input DNA in each sample. m^+^p^+^, n = 3; m^+^p^Δ4.8^, n = 3; m^+^p^Δ4.8^, n = 3; m^+^p^Δ*Ndn*^, n = 3; m^Δ4.8^p^Δ*Ndn*^, n = 3. (D) Schematic diagram of the *Mkrn3* promoter. Gene structure is shown at the top, where the white box represents the partial *Mkrn3* exon with the +1 as the transcriptional start site. A *Not*I site at +139 is indicated. Locations of CpG dinucleotides are shown as vertical bars. Two primer pairs (half-arrows) were used for nested PCR to amplify the bisulfite-treated DNA at the *Mkrn3* promoter from −469 to +91 (black line). A third primer pair (half-arrows) was used for the MeDIP-qPCR analysis to amplify the region from +21 to +324 (black line). (E) Sodium bisulfite sequencing analyses of methylation status of 22 CpG dinucleotides across the *Mkrn3* promoter (−469 to +91) in the wild-type m^+^p^+^ mice and the m^Δ4.8^p^+^ mice. Each line represents an individual clone with open and closed circles corresponding to unmethylated and methylated CpGs, respectively. (F) MeDIP-qPCR analyses of DNA methylation at the *Mkrn3* promoter (+21 to +324) in the wild-type m^+^p^+^ mice, the m^Δ4.8^p^+^ mice, and the m^+^p^Δ4.8^ mice. The level of MeDIP DNA was normalized against the level of input DNA in each sample. The normalized level of MeDIP DNA from the wild-type mouse was set as 1. m^+^p^+^, n = 3; m^+^p^Δ4.8^, n = 3; m^+^p^Δ4.8^, n = 3.

Next, we analyzed whether maternal inheritance of the Δ4.8 mutation affects the DNA methylation status at the PWS/AS domain. Silencing of the maternal alleles of *Snrpn*, *Ndn*, and *Mkrn3* is associated with maternal-specific CpG methylation on their promoters [33], [34], [35], [36]. Since the Δ4.8 mutation deletes the CpG island at the *Snrpn* promoter, we examined CpG methylation at the *Ndn* and *Mkrn3* loci. Consistent with an earlier report [6], Southern blot analysis using the methylation-sensitive *Sac*II enzyme revealed that CpG methylation on the *Sac*II site at the *Ndn* locus was not affected by maternal inheritance of the Δ4.8 mutation (Figure S2). However, use of a more sensitive analysis with sodium bisulfite sequencing revealed a lesser degree of methylation of the 42 CpGs on the *Ndn* promoter in the m^Δ4.8^p^+^ mice compared with that in wild-type mice (Figure 7B, top). Since CpG methylation at *Ndn* is maternal-specific [35], we further used mice with paternal inheritance of the Δ*Ndn* mutation to demonstrate maternal-specific reduction of CpG methylation. To enable detection of methylation only on the maternal *Ndn* allele, the reverse primers used in PCR to amplify the bisulfite-treated DNA were positioned within the region deleted in the Δ*Ndn* mutation (Figure 7A). Sodium bisulfite sequencing analyses showed decreased CpG methylation at the *Ndn* promoter on the maternal Δ4.8 chromosome in the m^Δ4.8^p^Δ*Ndn*^ mice (Figure 7B, bottom, right), when compared to the maternal wild-type chromosome in the m^+^p^Δ*Ndn*^ mice (Figure 7B, bottom, left). These results suggest that maternal inheritance of the Δ4.8 mutation decreased CpG methylation on the maternal *Ndn* allele.

Finally, methylated DNA immunoprecipitation with 5-methylcytidine specific antibody (MeDIP) followed by quantitative PCR analysis (MeDIP-qPCR) confirmed a reduction of methylated DNA in mice with maternal inheritance of the Δ4.8 mutation (m^Δ4.8^p^+^) (Figure 7C, left). In contrast, an increase of methylated DNA was found in mice with paternal inheritance of the Δ4.8 mutation (m^+^p^Δ4.8^) (Figure 7C, left), which is consistent with the previous report [6]. Using the primer pair located at the region deleted in the Δ*Ndn* mutation for MeDIP-qPCR analyses (Figure 7A), we confirmed maternal inheritance of Δ4.8 mutation contributes to reduction of DNA methylation at the maternal *Ndn* allele, when the m^Δ4.8^p^Δ*Ndn*^ mice was compare with the m^+^p^Δ*Ndn*^ mice (Figure 7C, right).

In addition to *Ndn*, we found that *Mkrn3* locus exhibited a similar alteration of DNA methylation by sodium bisulfite sequencing and MeDIP-qPCR analyses (Figure 7E and 7F), despite Southern blot analysis from an earlier report showed CpG methylation on the *Not*I site was not affected by maternal inheritance of the Δ4.8 mutation [6]. When sodium bisulfite sequencing were used to analyze 22 CpG sites at the *Mkrn3* promoter (Figure 7D), the m^Δ4.8^p^+^ mice showed a lesser degree of CpG methylation when compared with that in wild type mice (Figure 7E). Similarly, MeDIP-qPCR analyses demonstrated a reduction of methylated DNA in the m^Δ4.8^p^+^ mice (Figure 7F). In contrast, an increase of methylated DNA was found in the m^+^p^Δ4.8^mice (Figure 7F), which is consistent with the previous report [6]. These results suggested that maternal inheritance of the Δ4.8 mutation decreased DNA modification on *Ndn* and *Mkrn3*.

Taken together, maternal inheritance of the Δ4.8 mutation have a role in controlling allelic differential modifications at *Ndn* with increased H3K4me3 and H3Ac, decreased H3K9me3, and reduced DNA methylation, by which the maternal allele is changed toward a more paternal epigenotype. This was correlated with activation of the paternally expressed imprinted gene *Ndn* on the maternal chromosome by maternal inheritance of the Δ4.8 mutation (Figure 2).

## Discussion

Studies of the PWS-IC deletions in human patients and mouse models have suggested that the PWS-IC positively regulates paternal-specific gene expression and is responsible for establishment and maintenance of the paternal imprint [6], [20], [21]. On the other hand, it has been proposed that the AS-IC functions in allele-specific repression of the PWS-IC to prevent the paternal program on the maternal chromosome, and this interpretation is based on genetic analysis showing that the maternal AS-IC imposes a silent epigenetic state on the neighboring *SNRPN*/*Snrpn* promoter [22], [23], [24]. However, the equivalent of the human AS-IC element has not been identified in mice. In contrast to the current hypothesis that the PWS-IC as a positive regulator for the paternal imprinting program must be repressed on the maternal chromosome, our findings suggest another dimension that the PWS-IC is also required for a maternal chromosome to have the maternal pattern of gene expression. The maternal chromosome carrying the PWS-IC Δ4.8 mutation failed to properly silence the paternal imprinting program, suggesting that the maternal PWS-IC negatively regulates the paternally expressed imprinted genes, in stark contrast to its known function on the paternal chromosome as a positive regulator for paternal-specific gene expression.

**Figure 8:**
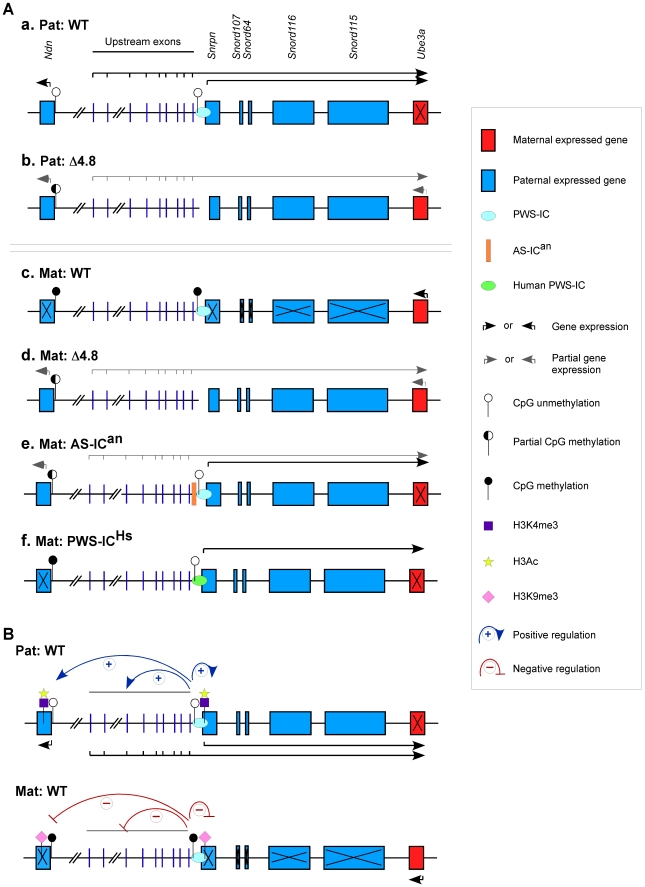
Schematic representation of genetic architecture at the PWS/AS domain. (A) Gene expression and DNA methylation associated with the Δ4.8 mutation, the AS-IC^an^ mutation, or the PWS-IC^Hs^ mutation were shown. On the paternal wild-type chromosome, the *Snrpn* and *Ndn* promoters are unmethylated and the paternally expressed imprinted genes are activated (a). When the PWS-IC Δ4.8 mutation deletes the CpG island at the *Snrpn* promoter on the paternal chromosome, the *Snrpn* sense/*Ube3a* antisense is transcribed only from the upstream exons, but is not transcribed from the major promoter *Snrpn* exon 1, resulting in partial activation of *Ube3a*. The *Ndn* promoter was partially methylated with decreased gene expression (b). On the maternal wild-type chromosome, silencing of *Ndn* and *Snrpn* is associated with DNA methylation at their promoters. *Ube3a* is activated (c). When the PWS-IC Δ4.8 mutation deletes the CpG island at the *Snrpn* promoter on the maternal chromosome, the *Snrpn* promoter at the upstream exons is activated and transcribes the *Snrpn* sense/*Ube3a* antisense, resulting in partial reduction of *Ube3a* expression. The *Ndn* promoter was partially activated with decreased DNA methylation (d). Maternal inheritance of an insertion/duplication mutation 13 kb upstream of *Snrpn* exon 1 (AS-IC^an^) causes loss of *Snrpn* methylation, decreased *Ndn* methylation, activation of the maternally repressed genes, and silencing of *Ube3a* due to expression of the maternal copy of the *Snrpn* sense/*Ube3a* antisense (e). Maternal transmission of a targeted replacement of mouse PWS-IC with human PWS-IC (PWS-IC^Hs^) expressed the *Snrpn* sense/*Ube3a* antisense transcripts from the inserted human *SNRPN* promoter, resulting in silencing of *Ube3a*. The PWS-IC^Hs^ does not affect any other paternally expressed imprinted transcripts on the maternal chromosome (f). (B) A model on how PWS-IC controls both paternal and maternal imprint at the PWS/AS domain. On the paternal chromosome, the PWS-IC functions as the major promoter for the *Snrpn* sense/*Ube3a* antisense transcripts. The paternal PWS-IC also acts at long distances to activate the *Snrpn* upstream exons and *Ndn* gene. The active *Snrpn* and *Ndn* promoters are unmethylated on the CpG islands and modified with H3K4me3 and H3Ac. On the other hand, the maternal PWS-IC acts *in cis* to silence the paternally expressed imprinted genes with the *Snrpn* and *Ndn* promoters methylated on the CpG islands and modified with H3K9me3, a mark of a repressive chromatin state. The maternally expressed imprinted genes *Ube3a* is expressed due to lack of the *Snrpn* sense/*Ube3a* antisense transcripts on the maternal chromosome.

The maternally expressed gene *UBE3A*/*Ube3a* is the AS gene and is negatively regulated by the paternal expressed *SNRPN* sense/*UBE3A* antisense and *Snrpn* sense/*Ube3a* antisense transcripts derived from the *SNRPN* and *Snrpn* promoters, respectively [14], [30]. On the wild-type maternal chromosome, silencing of the *Snrpn* promoter results in expression of *Ube3a* (Figure 8A, c). Previously, we demonstrated that maternal transmission of an insertion/duplication mutation 13 kb upstream of *Snrpn* exon 1 (AS-IC^an^, an anchor mutation on the AS-IC) activates the *Snrpn* promoter, resulting in severely decreased expression of *Ube3a*, causing AS phenotypes [26] (Figure 8A, e). In this report, we found that when the main *Snrpn* promoter was deleted, the maternal PWS-IC Δ4.8 mutation activates the weaker upstream alternative *Snrpn* promoter and expresses a low level of the *Snrpn* sense/*Ube3a* antisense transcripts, resulting in mild reduction of *Ube3a* expression (Figure 8A, d). Phenotype effects of the Δ4.8 mutation are being studied further for the symptoms of AS. In both cases of the Δ4.8 mutation and the AS-IC^an^ mutation, activation of paternally expressed imprinted genes on the maternal chromosome leads to the ability to complement the lethality and growth retardation phenotypes in mouse models of PWS. In addition, the acquisition the paternal gene expression pattern was correlated with alteration of DNA methylation on the maternal chromosome toward to a more paternal epigenotype: the AS-IC^an^ mutation causes loss of *Snrpn* methylation and decreased *Ndn* methylation (Figure 8A, e) and the Δ4.8 mutation causes decreased *Ndn* methylation on the maternal chromosome (Figure 8A, d), while the *Ndn* and *Snrpn* promoters are fully methylated on the maternal wild-type chromosome (Figure 8A, c)

The PWS-IC has a dual function, one as the *Snrpn* promoter and the other as an IC regulator of the PWS/AS domain. Maternal transmission of a targeted replacement of mouse PWS-IC with human PWS-IC (PWS-IC^Hs^) expressed the *Snrpn* sense/*Ube3a* antisense transcripts from the inserted human *SNRPN* promoter, but did not affect any other paternally expressed imprinted transcripts on the maternal chromosome (Figure 8A, f) [14], suggesting that the IC function was not lost. In our mouse model, maternal inheritance of the PWS-IC Δ4.8 mutation disrupts not only maternal imprinting of *Snrpn* but also maternal imprinting of *Ndn* which is 1 Mb away from the Δ4.8 region (Figure 8A, d), suggesting that this Δ4.8 mutation perturbs the IC function on the maternal imprint at the PWS/AS region. In addition, maternal inheritance of the PWS-IC^Hs^ rescues lethality in a PWS mouse model inheriting the PWS-IC 35-kb deletion (PWS-IC^del^) paternally, but the PWS-IC^Hs/del^ mice still have a growth deficiency [14], [20]. Maternal inheritance of the PWS-IC Δ4.8 mutation rescues both lethality and growth retardation phenotypes in PWS mouse models. The lethality and growth retardation phenotypes seem to correlate with the dual role of the PWS-IC as the *Snrpn* promoter and as the IC regulator for imprinted genes at the PWS/AS domain. Mouse models of PWS have failure to thrive which results in postnatal lethality and growth retardation. Maternal expression of the *Snrpn* sense/*Ube3a* antisense transcripts from the inserted human *SNRPN* promoter complements one failure to thrive locus to rescue lethality, but is not able to complement a second failure to thrive locus which contributes to a growth retardation phenotype [14]. In our mouse model, maternal inheritance of the PWS-IC Δ4.8 mutation perturbs the IC function of the maternal imprint at the PWS/AS region, and thereby activates the paternally expressed imprinted genes spread over the PWS/AS domain, which could complement all of the failure to thrive loci, resulting in rescues of both lethality and growth retardation phenotypes in PWS mouse models. Furthermore, maternal inheritance of the PWS-IC Δ4.8 mutation only caused a mild reduction of *Ube3a* expression (Figure 8A, d), whereas maternal inheritance of the PWS-IC^Hs^ resulted in severely decreased expression of *Ube3a* (Figure 8A, f), leading to AS phenotypes [14].

On the paternal wild-type chromosome, the *Snrpn* and *Ndn* promoters are unmethylated and the paternally expressed imprinted genes are fully activated (Figure 8A, a). When paternal inheritance of the Δ4.8 mutation deletes the *Snrpn* exon 1, the *Snrpn* sense/*Ube3a* antisense partially transcribes from the upstream exons (Figure 8A, b). Although mice with paternal inheritance of the Δ4.8 mutation expressed relatively similar levels of the *Snrpn*, *Snord116*, and *Snord115* transcripts as mice with maternal inheritance of the Δ4.8 mutation (Figure 8A, b and d), different phenotypic effects of the Δ4.8 mutation were found depending on the origin of inheritance: paternal transmission of the Δ4.8 mutation caused PWS phenotypes showing postnatal lethality and growth retardation [6]; maternal inheritance of the Δ4.8 mutation is able to complement postnatal lethality and growth retardation phenotypes in the PWS mouse models. These results raise a possibility that gene(s) other than *Snord116*, and *Snord115*, and *Snrpn* are also involved in these PWS phenotypes, although deficiency of *SNORD116* in human or *Snord116* in mice has been demonstrated to contribute to PWS pathogenesis [9], [10], [11], [12], [13]. This hypothesis is also supported by analyses of several mouse models for PWS [2], [15]. We noticed that the above parent-of-origin specific effects of the Δ4.8 mutation appeared to correlate with the levels of the *Ndn* transcripts, in the condition with *Snrpn*, *Snord116*, and *Snord115* expressed only from the upstream alternative *Snrpn* promoters. Specifically, the m^+^/p^Δ4.8^ mice showed partial expression of *Ndn* with 50% lethality and growth retardation [6], whereas the m^Δ4.8^/p^ΔS-U^ mice could express normal or increased levels of total *Ndn* transcripts and appeared phenotypically normal; both m^+^/p^Δ4.8^ and m^Δ4.8^/p^ΔS-U^ mice could express similar levels of *Snrpn*, *Snord116*, and *Snord115*. Although these results suggest that *Ndn* is also a potential candidate gene responsible for the PWS phenotypes, it should be pointed out that targeted deletions of *Ndn* in mice had reported contradictory results, ranging from no to severe effects on lethality [29], [37], [38]. The reason for the differences is not clear, genetic backgrounds are suspected to be a contributing factor. However, growth retardation has not been reported in surviving mice with *Ndn* deficiency [29], [37], [38]. Loss of another gene or more than one gene regulated by the maternal PWS-IC might contribute to the lethality and growth retardation phenotype. Two mouse models with different targeted mutations of *Magel2* have been created. The first study indicated reduced embryonic viability and postnatal growth retardation from birth until weaning [39]. The second study showed neonatal lethality (around 50%) and postnatal growth retardation due to the suckling deficit [40].

The partial imprinting defect caused by maternal or paternal inheritance of the PWS-IC Δ4.8 mutation indicates that one or more elements outside the Δ4.8 region are additionally required for full PWS-IC activity [6]. Recently, paternal transmission of a deletion extended 1 kb further upstream of the Δ4.8 region results in fully penetrant imprinting defects, suggesting that this 1-kb interval contains functional elements that confer full PWS-IC activity with the Δ4.8 region [25]. We found that maternal inheritance of the Δ4.8 mutation obtained H3K4me3 enrichment and reduced H3K9me3 located within this 1-kb region just upstream of the Δ4.8 mutation. These epigenetic changes are being studied further for their parent of origin and function as a potential IC or promoter. If present only on the maternal allele, it is possible that the maternal PWS-IC Δ4.8 mutation could activate the remaining portion of the PWS-IC by creating an active chromatin hub on the maternal chromosome. Thereby, partial expression of the paternally expressed imprinted genes on the maternal chromosome could be due to activation of this potential IC element or be the direct effects of a partial loss of the PWS-IC by the Δ4.8 mutation. On the other hand, when paternally inheriting the Δ4.8 mutation, H3K4me3 enrichment is not present at the PWS-IC, although the paternally expressed imprinted genes are also partially expressed with the remaining portion of the PWS-IC.

Together, our findings provide evidence for the first time that the PWS-IC functions not only in paternal imprinting but also in maternal imprinting at the PWS/AS domain in mice (Figure 8B). The PWS-IC controls expression of imprinted genes accompanied by parent-specific epigenetic modifications. On the paternal chromosome, the PWS-IC positively regulates the paternally expressed imprinted genes with the *Snrpn* and *Ndn* promoters are unmethylated on the CpG islands and modified with active chromatin marks H3K4me3 and H3Ac [41] (Figure 8B). On the maternal chromosome, the PWS-IC represses expression of the paternally expressed imprinted genes with the *Snrpn* and *Ndn* promoters are methylated on the CpG islands and modified with a repressive chromatin mark H3K9me3 (Figure 8B). Furthermore, we demonstrated PWS phenotypic rescues by maternal inheritance of the PWS-IC deletion, in contrast to paternal inheritance of the PWS-IC deletion causing the PWS phenotypes. We identified a previously unrecognized and important role of the PWS-IC at the PWS/AS domain.

## Materials and Methods

### Ethics statement

All of the mice were bred and maintained according to a protocol (protocol number: AN772) approved by the Baylor College of Medicine Animal Care and Use Committee at the institution's specific pathogen-free mouse facility, which is approved by the American Association for Accreditation of Laboratory Animal Care and is operated in accordance with current regulations and standards of the US Department of Agriculture and the Department of Health and Human Services.

### Mouse models

Mutant mice with a 4.8-kb deletion (Δ4.8) at *Snrpn* exon 1were generated as described [6]. Mutant mice carrying a deletion from *Snrpn* exon 2 to *Ube3a* (ΔS-U) were previously described [28]. Mutant mice with a deletion at *Ndn* (Δ*Ndn*) were described [29]. Mice with the Δ4.8 mutation and mice with the ΔS-U mutation are maintained on a C57BL6/J genetic background. Mice with the Δ*Ndn* mutation are maintained on a hybrid C57BL6/J and 129/SvEv genetic background.

### RT-PCR analysis

Total RNA was purified from mouse whole brain dissected from pups at day 1 of age using an RNeasy plus kit (Qiagen, Hilden, Germany). 2 µg of DNase I-treated total RNA was used for reverse transcription to synthesize the first strand cDNA by the Superscript III First-strand synthesis system (Invitrogen, Carlsbad, CA). Quantitative RT-PCR analysis was performed using LightCycler Fast-Start DNA Master SYBR Green I (Roche). PCR conditions and primer sequences are listed in Table S1 [6], [13], [26], [42]. *Hprt* and *Gapdh* transcripts were amplified as controls for gene expression. For quantification experiments, we used a least 3 sets of mice with every genotypes. In each group of mice with different genotypes, the levels of gene expression were normalized against the levels of an endogenous control in each sample. In each set of experiments, the normalized level of gene expression from the wild-type mouse was always set as 1.

### Western blot analysis

Mouse whole brain was dissected from pups at day 1 of age. Brain samples were homogenized and lysed in NP40/SDS buffer {1% Nonidet P-40, 0.01% SDS, 0.1 M Tris-HCl, pH 7.2, and complete Protease Inhibitor Cocktail Tablet (Roche Applied Sciences, Indianapolis IN)}. Sixty micrograms of mouse brain protein were used for electrophoresis on 10% Tris-Cl ready gels (Bio-Rad, Hercules CA). The proteins were transferred to nitrocellulose membrane (Bio-Rad). The membranes were then incubated with the appropriate antibodies as follows: rabbit anti-human E6-AP was diluted 1∶1000 (A300-352A; Bethyl Labs, Montgomery TX) or goat anti-human actin IgG was diluted 1∶500 (ac-1616 Santa-Cruz Biotechnology, Santa Cruz CA). The membranes were then incubated with either goat anti-rabbit IgG horseradish peroxidase (HRP) or donkey anti-goat HRP (ac-2004 or ac-2020, respectively, Santa Cruz). The signals of western blotting were detected by enhanced chemiluminesence (ECL, GE healthcare) exposed to X-ray films. For quantification experiments, the X-ray films were scanned and the intensity of the signals was quantified by densitometry. We used a least 3 sets of mice with every genotypes. In each group of mice from different genotypes, the levels of E6-AP were normalized against the levels of actin in each sample. In each set of experiments, the normalized level of E6-AP from the wild-type mouse was always set as 1.

### Chromatin immunoprecipitation (ChIP)

For ChIP-on-chip assays, mouse whole brain dissected from pups at day 1 of age was used for MNase chromatin immunoprecipitation assay as described [43]. Brain samples were homogenized in Douncing buffer {10 mM Tris-Cl at pH 7.5, 4 mM MgCl_2_, 2 mM CaCl_2_, and complete Protease Inhibitor Cocktail Tablet (Roche)}, treated with Micrococcal nuclease (0.006 unit/µl), and then lysed with hypotonic solution {0.1 M DTT, 0.1 M EDTA, 0.01 M PMSF, 0.1 M benzamidine, and complete Protease Inhibitor Cocktail Tablet (Roche)}. For chromatin modification analysis, chromatin was extracted in incubation buffer {0.1 M EDTA at pH 8.0, 0.1 M Tris-Cl, 0.1 M NaCl, and complete Protease Inhibitor Cocktail Tablet (Roche)}, and was immunoprecipitated with anti-trimethyl H3K4 antibodies (Abcam, clone ID: ab8580).

For ChIP-qPCR analyses, mouse whole brain dissected from pups at day 1 of age was used for ChIP assays as described by Millipore/Upstate Biotechnology (available at http://www.millipore.com). For chromatin modification analysis, chromatin extracted from mouse brain was immunoprecipitated with anti-trimethyl H3K4 antibodies (Abcam, clone ID: ab8580), anti-acetyl Histone 3 (Millipore/Upstate, catalog #: 06-599), or anti-trimethyl H3K9me3 (Millipore/Upstate, clone ID: 6F12-H4). Then, qPCR analyses were performed using the primer sets to amplify co-precipitated DNA from *Snrpn* and *Ndn*. The primers used are listed in Table S1.

### Mouse genomic tiling array

We designed a custom mouse genomic tiling array using the Agilent E-array platform. The array included 44,000 oligonucleotides covering sequences of the mouse imprinted gene clusters from *ATP10a* to *Peg12* at the PWS/AS region (Chr7: 64,846,543–69,740,076). DNA products from chromatin immunoprecipitation were labeled and applied to the genomic tiling arrays as described by the protocol of Agilent Technologies (available at http://www.agilent.com).

### Sodium bisulfite sequencing analysis

Genomic DNA was purified from mouse brain dissected from pups at day 1 of age using the DNeasy blood & Tissue Kit (Qiagen, Hilden, Germany). Bisulfite treatment of genomic DNA was carried out using the EZ DNA methylation kit (ZYMO Research, Irvin, CA) as described (available at http://www.zymoresearch.com). From bisulfite-treated DNA, the CpG-rich regions of *Ndn* and *Mkrn3* were amplified by nested PCR with primer sets listed in Table S1. For the *Ndn* CpG region, a 700-bp first-round PCR product was amplified with the forward primer NEC43F [44] and the reverse primer *Ndn*-bis1R. Then, a 628-bp second-round PCR product was amplified with the forward primer *Ndn*-bis2F and the reverse primer *Ndn*-bis2R. For the *Mkrn3* CpG region, a 625-bp first-round PCR product was amplified with the forward primer *Mkrn3*-bis1F and the reverse primer W28 [27]. Then, a 606-bp second-round PCR product was amplified with the forward primer *Mkrn3*-bis2F and the reverse primer *Mkrn3*-bis2R. Nested PCR reactions were performed with ZymoTaq DNA polymerase (ZYMO Research) with first-round PCR as follows: 10 min at 94°C; 1 min at 94°C, 2 min at 58°C, and 2 min at 72°C cycled 5 times; 45 s at 94°C, 1 min at 58°C, and 1 min at 72°C cycled 35 times; 10 min at 72°C, and second-round PCR as follows: 10 min at 94°C; 45 s at 94°C, 1 min at 58°C, and 1 min at 72°C cycled 35 times; 10 min at 72°C. PCR products from two independent bisulfite conversion reactions were cloned into the pGEM-Teasy vector (Promega Corp.). DNA sequencing was performed using forward and reverse primers T7 and SP6 (MCLAB, South San Francisco, CA).

### Methylated DNA immunoprecipitation (MeDIP)

Genomic DNA was purified from mouse brain dissected from pups at day 1 of age using the DNeasy blood & Tissue Kit (Qiagen, Hilden, Germany). MeDIP assays were performed as described [45]. Briefly, 5 µg of genomic DNA in MeDIP buffer (10 mM sodium phosphate at pH 7.0, 140 mM NaCl, 0.05% Triton X-100) was sonicated to produce random fragments ranging in size from 300 bp to 1,000 bp. The DNA was immunoprecipitated with antibody against 5-methylcytidine (Eurogentec, Belgium, clone ID: 33D3) at 4°C for overnight and washed with MeDIP buffer three times. The precipitated DNA was treated with proteinase K at 50°C for 4 h and recovered by QIAprep Spin Miniprep kit (Qiagen, Hilden, Germany). Then, qPCR analyses were performed to amplify the precipitated DNA from *Ndn* and *Mkrn3*. The primers used are listed in Table S1.

## Supporting Information

Figure S1 **Expression analyses of** ***Snrpn*** **,** ***Snrod116*** **, and** ***Snord115*** **in the m^Δ4.8^p^+^ mice.** (A–D) The *Snrpn* u1-ex3 (A), *Snrpn* exon 7 (B), *Snrod116* (C), and *Snord115* (D) transcripts were analyzed by qRT-PCR. Total RNA was isolated from brains of wild-type mice (a, m^+^p^+^) (n = 4) and mice inheriting the Δ4.8 mutation maternally (b, m^Δ4.8^p^+^) (n = 4). For qRT-PCR, the levels of gene expression from wild-type mice were set as 1. (E) Schematic representation of the mouse PWS/AS domain (top) and summary of gene expression in the m^+^p^+^ and m^Δ4.8^p^+^ mice (bottom, a and b). The *Snrpn* sense/*Ube3a* antisense transcripts initiated from *Snrpn* exon 1 with the major promoter activity and from *Snrpn* upstream exons with weaker promoter activity are marked as bold and thin arrows, respectively. SnoRNAs are encoded within these large *Snrpn* sense/*Ube3a* antisense transcripts derived from both *Snrpn* major and upstream exon promoters. *Snord116* and *Snord115* are multiple copy gene clusters. The centromeric (cen) and the telomeric (tel) positions are indicated. Paternally and maternally expressed genes are marked as blue and red boxes, respectively. Δ4.8 indicates a 4.8-kb deletion at *Snrpn* exon 1. The levels of the *Snrpn* u1-ex3, *Snrpn* exon 7, *Snrod116*, and *Snord115* transcripts from wild-type mice were set as 100%. Mat, maternal chromosome; Pat, paternal chromosome.
(TIF)

Figure S2 **Methylation analysis of** ***Ndn*** **in mice carrying the Δ4.8 deletion.** Southern blotting was performed to analyze the methylation patterns of the *Ndn* CpG island in mice with paternal inheritance of the Δ4.8 deletion (m^+^p^Δ4.8^), mice with maternal inheritance of the Δ4.8 deletion (m^Δ4.8^p^+^), mice with the Δ4.8 deletion from both parents (m^Δ4.8^p^Δ4.8^), and wild type mice (m^+^p^+^). Genomic DNA isolated from brains was digested with *Hin*dIII (H) alone or in combination with *Sac*II (SH), and hybridized with a probe from the *Ndn* 5′ flanking region. Fragment sizes: me, 3.4 kb; unme, 1.9 kb. me, methylated; unme, unmethylated.
(TIF)

Table S1 **Primer sequences and conditions for PCR.**
(XLS)

## References

[1] Buiting K (2010). Prader-Willi syndrome and Angelman syndrome.. Am J Med Genet C Semin Med Genet.

[2] Relkovic D, Isles AR (2011). Behavioural and cognitive profiles of mouse models for Prader-Willi syndrome.. Brain Res Bull.

[3] Gray TA, Saitoh S, Nicholls RD (1999). An imprinted, mammalian bicistronic transcript encodes two independent proteins.. Proc Natl Acad Sci U S A.

[4] Dittrich B, Buiting K, Korn B, Rickard S, Buxton J (1996). Imprint switching on human chromosome 15 may involve alternative transcripts of the SNRPN gene.. Nat Genet.

[5] Farber C, Dittrich B, Buiting K, Horsthemke B (1999). The chromosome 15 imprinting centre (IC) region has undergone multiple duplication events and contains an upstream exon of SNRPN that is deleted in all Angelman syndrome patients with an IC microdeletion.. Hum Mol Genet.

[6] Bressler J, Tsai TF, Wu MY, Tsai SF, Ramirez MA (2001). The SNRPN promoter is not required for genomic imprinting of the Prader-Willi/Angelman domain in mice.. Nat Genet.

[7] Landers M, Bancescu DL, Le Meur E, Rougeulle C, Glatt-Deeley H (2004). Regulation of the large (approximately 1000 kb) imprinted murine Ube3a antisense transcript by alternative exons upstream of Snurf/Snrpn.. Nucleic Acids Res.

[8] Runte M, Huttenhofer A, Gross S, Kiefmann M, Horsthemke B (2001). The IC-SNURF-SNRPN transcript serves as a host for multiple small nucleolar RNA species and as an antisense RNA for UBE3A.. Hum Mol Genet.

[9] Sahoo T, del Gaudio D, German JR, Shinawi M, Peters SU (2008). Prader-Willi phenotype caused by paternal deficiency for the HBII-85 C/D box small nucleolar RNA cluster.. Nat Genet.

[10] de Smith AJ, Purmann C, Walters RG, Ellis RJ, Holder SE (2009). A deletion of the HBII-85 class of small nucleolar RNAs (snoRNAs) is associated with hyperphagia, obesity and hypogonadism.. Hum Mol Genet.

[11] Duker AL, Ballif BC, Bawle EV, Person RE, Mahadevan S (2010). Paternally inherited microdeletion at 15q11.2 confirms a significant role for the SNORD116 C/D box snoRNA cluster in Prader-Willi syndrome.. Eur J Hum Genet.

[12] Skryabin BV, Gubar LV, Seeger B, Pfeiffer J, Handel S (2007). Deletion of the MBII-85 snoRNA gene cluster in mice results in postnatal growth retardation.. PLoS Genet.

[13] Ding F, Li HH, Zhang S, Solomon NM, Camper SA (2008). SnoRNA Snord116 (Pwcr1/MBII-85) deletion causes growth deficiency and hyperphagia in mice.. PLoS One.

[14] Johnstone KA, DuBose AJ, Futtner CR, Elmore MD, Brannan CI (2006). A human imprinting centre demonstrates conserved acquisition but diverged maintenance of imprinting in a mouse model for Angelman syndrome imprinting defects.. Hum Mol Genet.

[15] Chamberlain SJ, Lalande M (2010). Neurodevelopmental disorders involving genomic imprinting at human chromosome 15q11–q13.. Neurobiol Dis.

[16] Kishino T, Lalande M, Wagstaff J (1997). UBE3A/E6-AP mutations cause Angelman syndrome.. Nat Genet.

[17] Matsuura T, Sutcliffe JS, Fang P, Galjaard RJ, Jiang YH (1997). De novo truncating mutations in E6-AP ubiquitin-protein ligase gene (UBE3A) in Angelman syndrome.. Nat Genet.

[18] Cattanach BM, Barr JA, Beechey CV, Martin J, Noebels J (1997). A candidate model for Angelman syndrome in the mouse.. Mamm Genome.

[19] Jiang YH, Armstrong D, Albrecht U, Atkins CM, Noebels JL (1998). Mutation of the Angelman ubiquitin ligase in mice causes increased cytoplasmic p53 and deficits of contextual learning and long-term potentiation.. Neuron.

[20] Yang T, Adamson TE, Resnick JL, Leff S, Wevrick R (1998). A mouse model for Prader-Willi syndrome imprinting-centre mutations.. Nat Genet.

[21] Bielinska B, Blaydes SM, Buiting K, Yang T, Krajewska-Walasek M (2000). De novo deletions of SNRPN exon 1 in early human and mouse embryos result in a paternal to maternal imprint switch.. Nat Genet.

[22] Ohta T, Buiting K, Kokkonen H, McCandless S, Heeger S (1999). Molecular mechanism of angelman syndrome in two large families involves an imprinting mutation.. Am J Hum Genet.

[23] Shemer R, Hershko AY, Perk J, Mostoslavsky R, Tsuberi B (2000). The imprinting box of the Prader-Willi/Angelman syndrome domain.. Nat Genet.

[24] Perk J, Makedonski K, Lande L, Cedar H, Razin A (2002). The imprinting mechanism of the Prader-Willi/Angelman regional control center.. EMBO J.

[25] Dubose AJ, Smith EY, Yang TP, Johnstone KA, Resnick JL (2011). A new deletion refines the boundaries of the murine Prader-Willi syndrome imprinting center.. Hum Mol Genet.

[26] Wu MY, Chen KS, Bressler J, Hou A, Tsai TF (2006). Mouse imprinting defect mutations that model Angelman syndrome.. Genesis.

[27] Peery EG, Elmore MD, Resnick JL, Brannan CI, Johnstone KA (2007). A targeted deletion upstream of Snrpn does not result in an imprinting defect.. Mamm Genome.

[28] Tsai TF, Jiang YH, Bressler J, Armstrong D, Beaudet AL (1999). Paternal deletion from Snrpn to Ube3a in the mouse causes hypotonia, growth retardation and partial lethality and provides evidence for a gene contributing to Prader-Willi syndrome.. Hum Mol Genet.

[29] Tsai TF, Armstrong D, Beaudet AL (1999). Necdin-deficient mice do not show lethality or the obesity and infertility of Prader-Willi syndrome.. Nat Genet.

[30] Rougeulle C, Cardoso C, Fontes M, Colleaux L, Lalande M (1998). An imprinted antisense RNA overlaps UBE3A and a second maternally expressed transcript.. Nat Genet.

[31] Chamberlain SJ, Brannan CI (2001). The Prader-Willi syndrome imprinting center activates the paternally expressed murine Ube3a antisense transcript but represses paternal Ube3a.. Genomics.

[32] Lau JC, Hanel ML, Wevrick R (2004). Tissue-specific and imprinted epigenetic modifications of the human NDN gene.. Nucleic Acids Res.

[33] Shemer R, Birger Y, Riggs AD, Razin A (1997). Structure of the imprinted mouse Snrpn gene and establishment of its parental-specific methylation pattern.. Proc Natl Acad Sci U S A.

[34] Gabriel JM, Gray TA, Stubbs L, Saitoh S, Ohta T (1998). Structure and function correlations at the imprinted mouse Snrpn locus.. Mamm Genome.

[35] Jay P, Rougeulle C, Massacrier A, Moncla A, Mattei MG (1997). The human necdin gene, NDN, is maternally imprinted and located in the Prader-Willi syndrome chromosomal region.. Nat Genet.

[36] Hershko A, Razin A, Shemer R (1999). Imprinted methylation and its effect on expression of the mouse Zfp127 gene.. Gene.

[37] Gerard M, Hernandez L, Wevrick R, Stewart CL (1999). Disruption of the mouse necdin gene results in early post-natal lethality.. Nat Genet.

[38] Muscatelli F, Abrous DN, Massacrier A, Boccaccio I, Le Moal M (2000). Disruption of the mouse Necdin gene results in hypothalamic and behavioral alterations reminiscent of the human Prader-Willi syndrome.. Hum Mol Genet.

[39] Bischof JM, Stewart CL, Wevrick R (2007). Inactivation of the mouse Magel2 gene results in growth abnormalities similar to Prader-Willi syndrome.. Hum Mol Genet.

[40] Schaller F, Watrin F, Sturny R, Massacrier A, Szepetowski P (2010). A single postnatal injection of oxytocin rescues the lethal feeding behaviour in mouse newborns deficient for the imprinted Magel2 gene.. Hum Mol Genet.

[41] Horsthemke B, Wagstaff J (2008). Mechanisms of imprinting of the Prader-Willi/Angelman region.. Am J Med Genet A.

[42] MacDonald HR, Wevrick R (1997). The necdin gene is deleted in Prader-Willi syndrome and is imprinted in human and mouse.. Hum Mol Genet.

[43] Dindot SV, Person R, Strivens M, Garcia R, Beaudet AL (2009). Epigenetic profiling at mouse imprinted gene clusters reveals novel epigenetic and genetic features at differentially methylated regions.. Genome Res.

[44] Hanel ML, Wevrick R (2001). Establishment and maintenance of DNA methylation patterns in mouse Ndn: implications for maintenance of imprinting in target genes of the imprinting center.. Mol Cell Biol.

[45] Weber M, Davies JJ, Wittig D, Oakeley EJ, Haase M (2005). Chromosome-wide and promoter-specific analyses identify sites of differential DNA methylation in normal and transformed human cells.. Nat Genet.

