# Supplementary figures and images for: An Unexpected Function of the Prader-Willi Syndrome Imprinting Center in Maternal Imprinting in Mice
Source: PLoS One. 2012 Apr 4;7(4):e34348. doi: 10.1371/journal.pone.0034348 (PMC3319576; doi:10.1371/journal.pone.0034348)

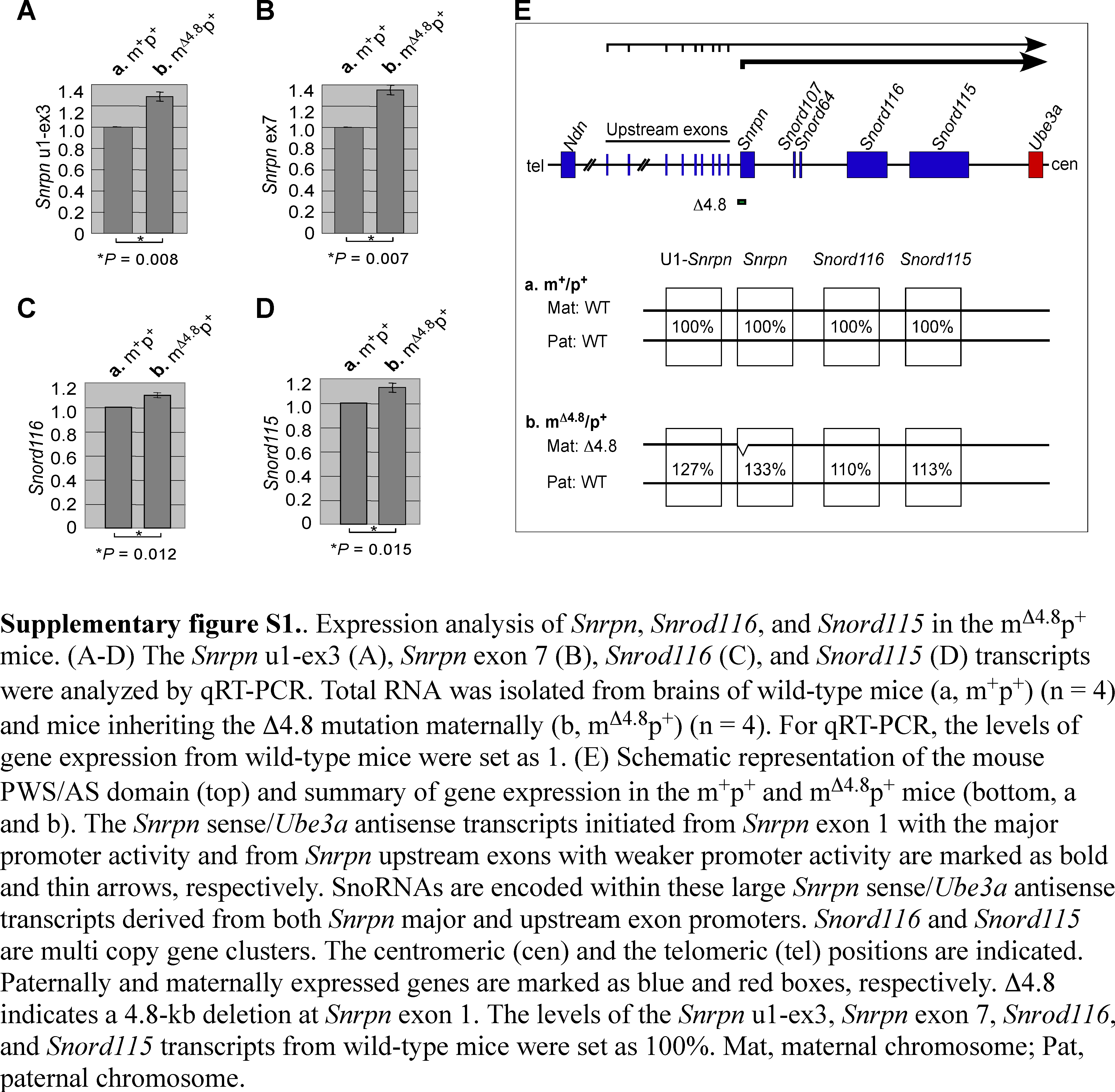

Supplement: Figure S1 — Expression analyses of Snrpn , Snrod116 , and Snord115 in the mΔ4.8p+ mice. (A–D) The Snrpn u1-ex3 (A), Snrpn exon 7 (B), Snrod116 (C), and Snord115 (D) transcripts were analyzed by qRT-PCR. Total RNA was isolated from brains of wild-type mice (a, m+p+) (n = 4) and mice inheriting the Δ4.8 mutation maternally (b, mΔ4.8p+) (n = 4). For qRT-PCR, the levels of gene expression from wild-type mice were set as 1. (E) Schematic representation of the mouse PWS/AS domain (top) and summary of gene expression in the m+p+ and mΔ4.8p+ mice (bottom, a and b). The Snrpn sense/Ube3a antisense transcripts initiated from Snrpn exon 1 with the major promoter activity and from Snrpn upstream exons with weaker promoter activity are marked as bold and thin arrows, respectively. SnoRNAs are encoded within these large Snrpn sense/Ube3a antisense transcripts derived from both Snrpn major and upstream exon promoters. Snord116 and Snord115 are multiple copy gene clusters. The centromeric (cen) and the telomeric (tel) positions are indicated. Paternally and maternally expressed genes are marked as blue and red boxes, respectively. Δ4.8 indicates a 4.8-kb deletion at Snrpn exon 1. The levels of the Snrpn u1-ex3, Snrpn exon 7, Snrod116, and Snord115 transcripts from wild-type mice were set as 100%. Mat, maternal chromosome; Pat, paternal chromosome. (TIF) [file pone.0034348.s001.tif]

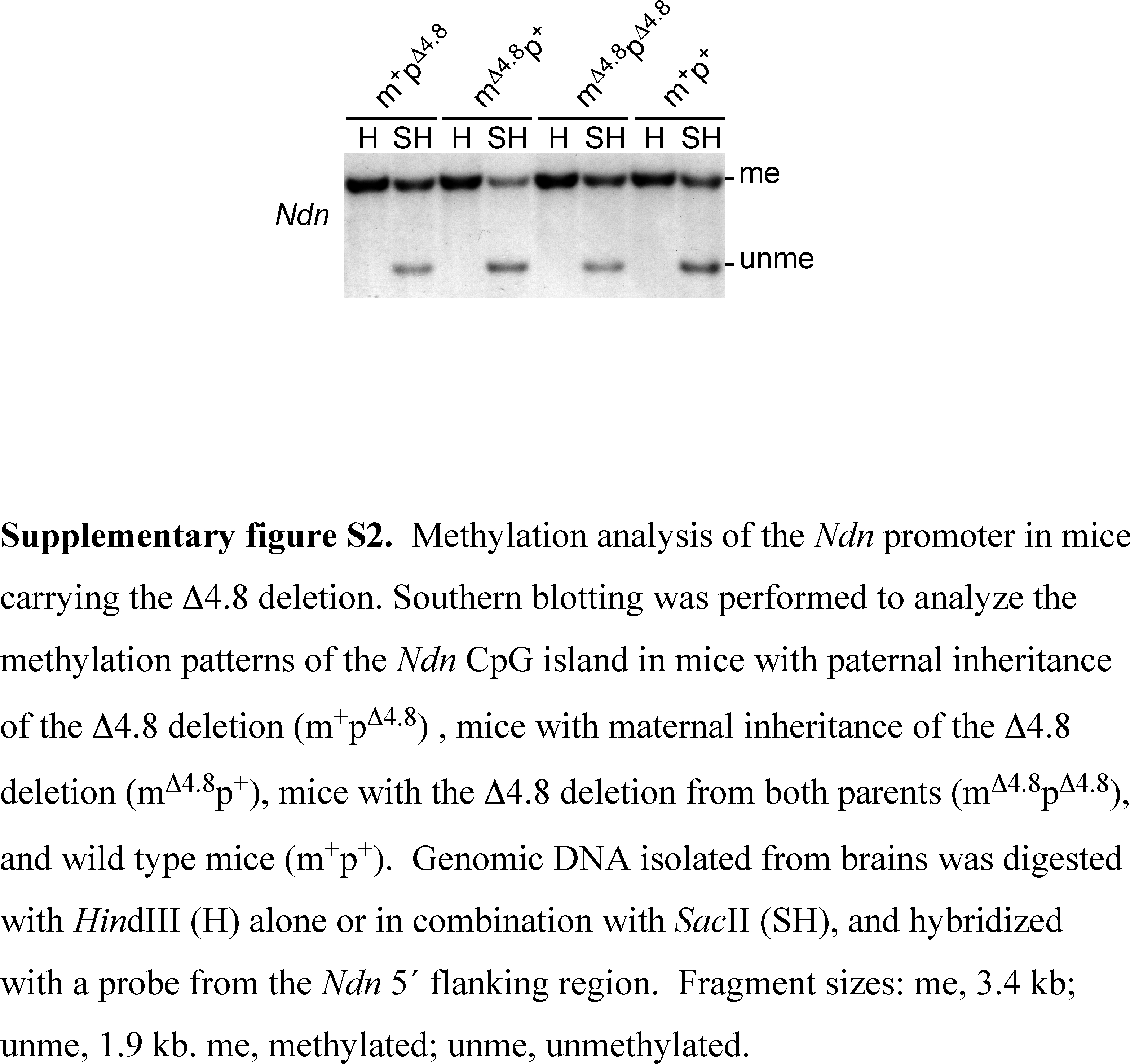

Supplement: Figure S2 — Methylation analysis of Ndn in mice carrying the Δ4.8 deletion. Southern blotting was performed to analyze the methylation patterns of the Ndn CpG island in mice with paternal inheritance of the Δ4.8 deletion (m+pΔ4.8), mice with maternal inheritance of the Δ4.8 deletion (mΔ4.8p+), mice with the Δ4.8 deletion from both parents (mΔ4.8pΔ4.8), and wild type mice (m+p+). Genomic DNA isolated from brains was digested with HindIII (H) alone or in combination with SacII (SH), and hybridized with a probe from the Ndn 5′ flanking region. Fragment sizes: me, 3.4 kb; unme, 1.9 kb. me, methylated; unme, unmethylated. (TIF) [file pone.0034348.s002.tif]
